# Comparing outcomes of Aquablation versus holmium laser enucleation of prostate in the treatment of benign prostatic hyperplasia: A network meta‐analysis

**DOI:** 10.1002/bco2.454

**Published:** 2024-10-30

**Authors:** Ansh Bhatia, Renil Titus, Joao G. Porto, Rajvi Goradia, Khushi Shah, Diana Lopategui, Thomas R. W. Herrmann, Hemendra N. Shah

**Affiliations:** ^1^ Miller School of Medicine Desai Sethi Urology Institute, University of Miami Miami Florida USA; ^2^ Seth GS Medical College and KEM Hospital Mumbai India; ^3^ Urology Spital Thurgau AG (STGAG) Frauenfeld Switzerland

**Keywords:** Aquablation, benign prostatic hyperplasia, holmium laser enucleation, lower urinary tract symptoms, TURP

## Abstract

**Introduction:**

Water Jet Ablation Therapy (WJAT) and Holmium Laser Enucleation of the Prostate (HoLEP) represent two common surgical treatments for Benign Prostatic Hyperplasia (BPH). Despite their increasing use, there is no study between these two methods. We aim to evaluate their efficacy and safety through a network meta‐analysis (NMA), providing critical insights for clinical decision‐making in the management of moderate to severe lower urinary tract symptoms (LUTS) due to BPH.

**Methods:**

Pubmed, EMBASE and Cochrane Library were searched. Randomized controlled trials and prospective single‐arm studies comparing WJAT and HoLEP with TURP, reporting symptom scores, flow rates and adverse events. Data extraction and quality assessments were independently performed. Bayesian modelling in RStudio was used for statistical analysis, evaluating continuous outcomes through mean difference and categorical variables via risk ratios. Risk‐of‐Bias (RoB) and GRADE assessments were performed.

**Findings:**

Twenty‐three studies were included (WJAT‐11, HoLEP‐12). Most studies were at some or high risk of bias. At 12 months, the IPSS, Qol, PVR and Qmax improvements were 4.14 points (95% CI: ‐0.34 to 8.64, not‐significant [NS], GRADE‐rating: Low), 0.32‐points (95% CI:‐10.70 to 3.27, NS, GRADE‐rating: Low), 2.45 ml/s (95% CI: ‐1.85 to 7.05, NS, GRADE‐rating: Low), 63.10 ml (95% CI: 39.80 to 87.30, statistically‐significant [SS], GRADE‐rating: Moderate), respectively, all in favour of HoLEP. Haemoglobin‐loss was lower with HoLEP, 1.16 g/dl (95% CI: ‐2.56 to 0.54 mg/dl, NS, GRADE‐rating: Moderate) than WJAT. The risk of incontinence was higher with HoLEP; 4.48 (95% CI: 0.22 to 168.50, NS, GRADE‐rating: Very Low) than WJAT in single–arm analysis. The risk of blood transfusion was higher with WJAT (RR = 0.14; 95% CI: 0.00 to 4.21, NS, GRADE‐rating: Low) than HoLEP. Risk of Total Serious Adverse Events (Clavien‐Dindo grade>3) was higher with HoLEP (RR = 1.12, higher with HoLEP, 95% CI: 0.20 to 12.71, NS, GRADE‐rating: Low) than WJAT. Retreatment was lower with HoLEP (RR = 0.46, 95% CI: 0.02 to 10.54 GRADE‐rating: Low) than WJAT.

**Interpretation:**

Our study suggests that both HoLEP and WJAT are effective treatments for BPH, both with similar IPSS and QoL improvements. HoLEP excels in functional outcomes, particularly in improving Qmax and PVR. Conversely, WJAT, with its shorter operation time and hospital stays, presents a compelling alternative, particularly for outpatient settings.

## 1 INTRODUCTION

Benign prostatic hyperplasia (BPH) related to lower urinary tract symptoms (LUTS) is the most common urological cause of impairment to men's quality of life globally. The second highest source of years lived with disease worldwide was BPH.^1^ Consequently, the global cost of BPH treatment is over $78 billion annually.^2^ While the earlier stages of an enlarged prostate can be managed with medication, surgical treatment is the most effective solution for LUTS.^3^ Historically, transurethral resection of the prostate (TURP) has been the gold standard, but its high rate of sexual side effects and reduced safety for larger prostate continue to be a significant limiting factor.^4^, ^5^ Consequently, various procedures have been developed in recent decades to overcome TURP's limitations.

Over the last two decades, holmium laser enucleation of the prostate (HoLEP) has emerged as a new contender for the surgical gold standard for BPH treatment, showing similar efficacy but a better safety profile when compared to TURP and open simple prostatectomy.^6^, ^7^, ^8^ Consistent and safe outcomes across a wide range of prostate sizes make HoLEP a size‐independent procedure for enlarged glands.^3^, ^9^ However, its steep learning curve has been a major limiting factor in the widespread adaptation of this technique.^3^ Analysis of American Board of Urology case log data from 2008 to 2021 revealed that the use of HoLEP did not change over time.^10^

Among the newer procedures, Water Jet Ablation Therapy (WJAT), commonly known as Aquablation, has been gaining traction in the United States in the last decade, promising improvement similar to TURP while being safer for larger prostate volumes (PV).^11^ WJAT is also claimed to have significantly lower adverse events (AE), such as lower incontinence rates and sexual side effects.^12^ Similar to HoLEP, WJAT is also claimed to be a size‐independent procedure. However, even though both EAU and AUA guidelines appear to limit WJAT to patients with prostate sizes < 80 g,^13^, ^14^ the CAU frames a weak conditional recommendation endorsing WJAT as a treatment option for larger prostates up to 150 g.^15^ Similarly, in the real world, WJAT is offered to larger prostates.^16^ According to the latest AUA and EAU guidelines, Endoscopic enucleation of the prostate is the preferred technique for these prostates.^13^, ^14^ Although HoLEP and WJAT are used for similar clinical indications and are vying for the mantle of the surgical gold standard in the treatment of BPH there is a notable absence of studies that compare these two modalities, including indirect comparisons. In this backdrop, we believe that an indirect comparison via a Network Meta‐Analysis (NMA) can provide a reasonable approach to provide information to urologists to counsel patients and help them make an informed decision via shared decision‐making.

Therefore, we conducted a network meta‐analysis (NMA) to provide an assessment of these size‐independent therapies, offering valuable insights for both practitioners and patients. We aim to answer the following question: Is there a clinically significant difference in the safety and efficacy outcomes between WJAT and HoLEP for surgical management of men with moderate to severe LUTS due to BPH?

## 2 METHODS AND MATERIALS

This NMA was registered to the PROSPERO database (CRD42023413783) and was conducted following the PRISMA‐N guidelines. Population, Intervention, Comparison and Outcomes (PICOs) criteria was used for formulating the research question and study selection (Table 1).

**TABLE 1:** PICOs criteria for this NMA.

Participants	Intervention	Comparator	Outcomes*	Description of Outcome
Adult males with moderate/severe LUTS due to BPH	WJAT HoLEP	TURP	International Prostate Symptom Score (IPSS)	Assesses symptoms based on patient' perspective. Scored from 0‐35
			IPSS‐Quality of Life Score (QoL)	Measure the impact of BPH symptoms on patient's quality of life. Scored from 0‐6.
			Post‐void Residual volume (PVR)	Urodynamic metric which measures urine (ml) left in the bladder after an average micturition.
			Maximum urinary flow velocity (Qmax)	Urodynamic metric which determines maximum urinary flow velocity achieved in micturition.
			Serious Adverse effects (Dindo‐Clavien Classification grade ≥ 3)	Adverse effects which require surgical, radiological or endoscopic interventions for correction (15).

### 2.1 Search strategy

A search of Medline, EMBASE and the Cochrane‐library databases was conducted from inception to October 2023 with keywords and variations such as “HoLEP,” “Holmium Laser Enucleation,” “Aquablation”, “WJAT,” “Waterjet Ablation Therapy,” “Enlarged Prostate” and “Benign Prostatic Hyperplasia” with Boolean operators. The search strategy can be found in the supplemental dataset. Additional studies were searched for by going through the reference lists of the available articles.

### 2.2 Inclusion criteria and study selection

The title/abstract screening was performed in duplicate and independently by two team members (AB and RT). We did not include unpublished studies, and non‐English language studies which could be reasonably interpreted were searched for, but we did not find any such studies in our target databases. We included randomized controlled trials (RCT) and prospective single‐arm (non‐comparative) studies reporting the effects of HoLEP and WJAT compared to TURP with at least one of the following outcome variables: International Prostate Symptom Score (IPSS), postvoid residual volume (PVR), quality of life (QoL), maximum peak flow rate (Qmax), retreatment rate (RTR) and serious adverse events (SAE) rates at least one of the following time points: 3, 6, 12, 24–36 and 60 months. AE of interest, such as acute urinary retention (AUR), urethral stricture and urinary tract infection (UTI) were also collected. Clinical heterogeneity (i.e., between‐trials differences in patients, treatments, outcomes characteristics, etc.) across eligible studies was assessed by evaluating participant and baseline characteristics.

**FIGURE 1:**
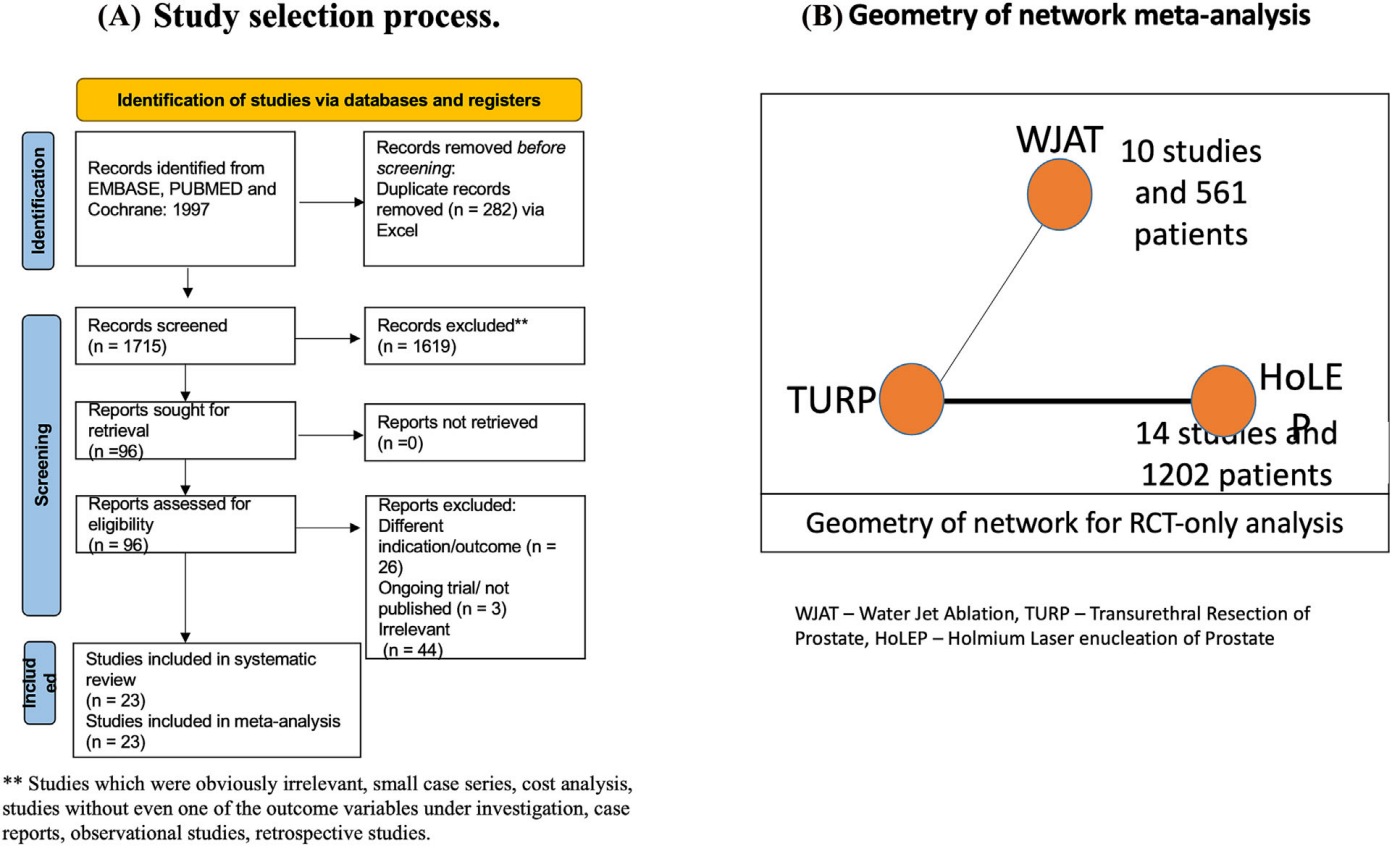
(A) PRISMA study selection process for this NMA. (B) Geometry of network meta‐analysis showing comparator between WJAT and TURP.

Two sets of quantitative analyses were conducted to assess available data to prevent pooling different study designs. One set only pooled RCT outcomes, while the second set included prospective single‐arm studies without a critical risk of bias (RoB). We excluded studies that lacked the outcome variables being investigated, had different treatment indications, focused solely on cost analysis or were case studies. The study selection process is depicted in Figure 1A. After the search results were exported to MS Excel, duplicates were removed and the remaining studies were screened based on the title and abstract. A full‐text review was done by three authors (AB, RT and RG), and disagreement was resolved with consensus and input of a fourth member (JGP).

**FIGURE 2:**
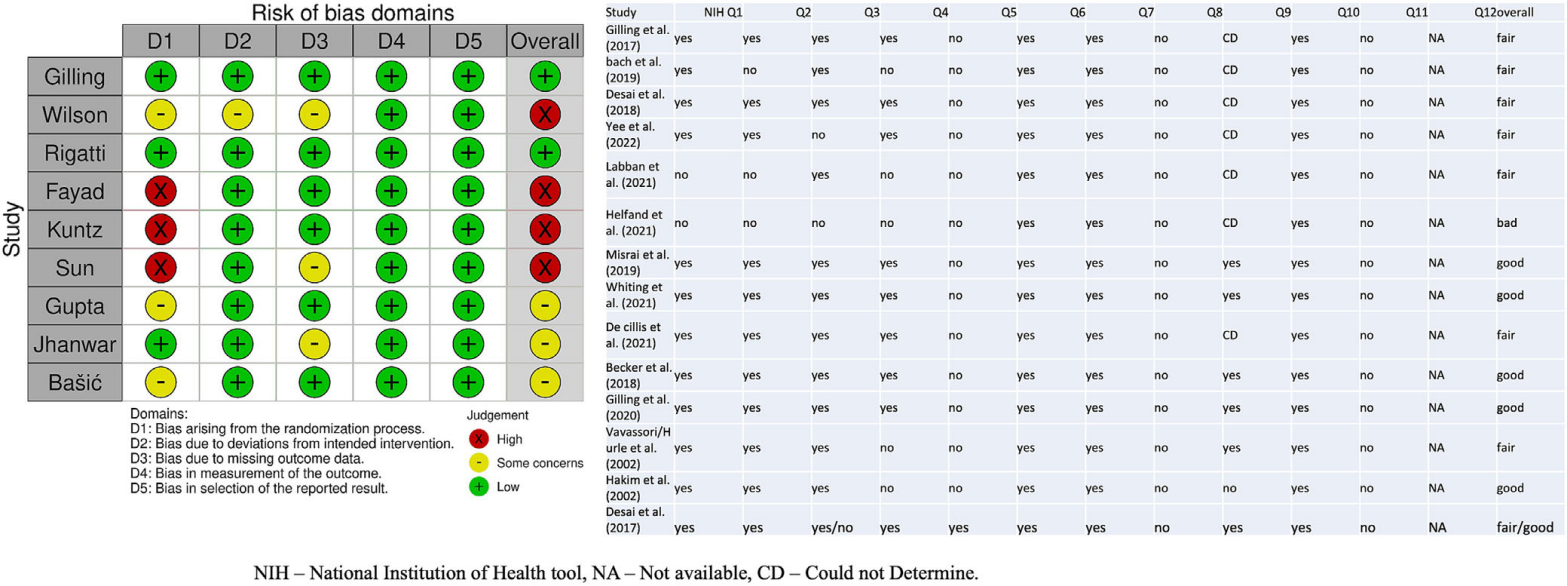
Risk of bias evaluation for included studies.

Three authors independently (RT, AB and RG) extracted the data from the selected studies, and the extraction was cross‐verified. Data points for 3, 12, 24–36 and 60 months were utilized for quantitative synthesis of early postoperative, short‐, mid‐ and long‐term follow‐up, respectively. If the data for these time points were unavailable, the closest follow‐up data was used. The geometry of the network is represented in Figure 1B. Two authors (AB and RT) did a RoB assessment using Cochrane's RoB‐2 assessment tool for RCTs and the NIH quality assessment tool for single‐arm (cohort) studies (Figure 2).^17^ Grading of Recommendations, Assessment, Development, and Evaluations (GRADE) criteria were used to assess the strength of evidence.^18^

### 2.3 Statistical analysis

The data was analysed using RStudio Version 4.3.3 (RStudio, Boston, MA). Bayesian modelling was used for this NMA. To estimate the effect size, continuous outcomes were analysed using mean difference (MD) with 95% credible intervals (CI). Categorical variables such as SAE and RTR were compared as risk ratios. An arm‐based NMA was performed to compare WJAT with HoLEP, an approach used extensively in the literature using the RStudio package “pcnetmeta”^19^, ^20^, ^21^, ^22^. Treatments were considered statistically superior if the 95% CI did not cross the line of no effect. SAE (≥ Clavien‐Dindo 3) and RTR were considered with the last available number of patients at the time of interest. NMA was performed separately for each outcome to determine the treatment effect estimate. Sensitivity analysis was performed by sequentially excluding studies at High RoB (Table S1). Funnel plots were evaluated for publication bias when more than 10 studies were included in the analysis.

## 3 RESULTS

### 3.1 Qualitative analysis

A total of 23 studies with 1626 patients were included: 11 studies including 662 patients for WJAT,^12^, ^23^, ^24^, ^25^, ^26^, ^27^, ^28^, ^29^, ^30^, ^31^, ^32^ 12 studies with 1204 patients for HoLEP.^33^, ^34^, ^35^, ^36^, ^37^, ^38^, ^39^, ^40^, ^41^, ^42^, ^43^, ^44^ The articles included in the WJAT analysis were 10 prospective single‐arm studies and one RCT. Eight RCTs (519 patients) and four prospective single‐arm studies (685 patients) were included for HoLEP (Table 2A and 2B).

**TABLE 2A:** Baseline characteristics of RCT studies.

Study name (country)	Type of study	Sample size	Imaging modality	HoLEP		TURP	
				Age	Prostate volume	Age	Prostate volume (mL)
Sun, 2014 (China)	RCT	164	TRUS	72.16 ± 7.53	55.11 ± 29.03	72.16 ± 7.53	56.22 ± 30.48
		82 (HoLEP), 82 (TURP)					
Fayad, 2015 (Egypt)	RCT	120	TRUS	60.85 ± 4.02	68.15 ± 11.16718	60.35 ± 3.926	67.2 ± 9.71945
		60 (HoLEP), 60 (TURP)					
		14 (HoLEP), 17 (TURP)					
Kuntz, 2004 (Germany)	RCT	200	TRUS	68.0 ± 7.3	53.5 ± 20.0	68.7 ± 8.2	49.9 ± 21.1
		100 (HoLEP), 100 (TURP)					
Gupta, 2005 (India)	RCT	150	TAUS	65.88 ± 10.1	57.9 ± 17.6	65.67 ± 7.5	59.8 ± 16.5
		50 (HoLEP), 100 (TURP)					
Montorsi, Rigatti, 2004 (Italy)	RCT	100	TRUS	65.14	70.3 ± 36.7	64.5	56.2 ± 19.4
		52 (HoLEP), 48 (TURP)					
Tan, 2003/Liam, 2006/Gilling 2011 (New Zealand)	RCT	61	TRUS	71.7 ± 1.1	77.8 ± 5.6	70.3 ± 1	70 ± 5
		31 (HoLEP), 30 (TURP)					
Basic, 2013 (Serbia)	RCT	40	TRUS	63.3 ± 7.4	48.8 ± 4.9	65.1 ± 6.9	42.6 ± 4.4
		20 (HoLEP), 20 (TURP)					
Jhanwar, 2016 (India)	RCT	144	TRUS	67.70 ± 7.44	75.6 ± 12.84	66.78 ± 7.81	74.5 ± 12.56
		72 (HoLEP), 72 (TURP)					
Gilling, 2018 (Multi‐center)	RCT (WJAT vs TURP)	184	TRUS	66.0 ± 7.3 (WJAT)	54.1 ± 16.2	65.8 ± 7.2	51.8 ± 13.8
		117 (WJAT), 67 (TURP)	TRUS				

**TABLE 2b:** Baseline characteristics of single‐arm studies.

Study name (country)	Type of study	Procedure	Sample size	Age	Prostate volume
Gilling et al. (2017) (Multi‐country)	Single arm prospective	WJAT	21	70 ± 5	57.2 (30–102)
Labban et al. (2021) (Lebanon)	Single arm prospective	WJAT	59	68.3 (7.9)	71.4 (31.3)
Misrai et al. (2019) (France)	Single arm prospective	WJAT	30	68 (61– 72) x	68 (61– 72) 60 (45–69)
Whiting et al. (2021), (UK)	Single arm prospective	WJAT	55	64.1 ± 7.9	58.2 ± 23.9
Bhojani et al. (2019), (Multi‐center)	Single arm prospective	WJAT	101	100 cm3 : 67 ± 6.2	120.9 ± 15.1
Desai et al. (2018), India	Single arm prospective	WJAT	47	66 ± 6	48 ± 24
Bach et al. (2018), Germany	Single arm prospective	WJAT	118	69 (8; 88–52)	64.3 (32; 20– 154)
Yee et al. (2022), China	Single arm prospective	WJAT	20	60.8 ± 15.	66.4 ± 4.4
Helfand et al. (2021), USA	Single arm prospective	WJAT	34	69 ± 8	209 ± 56
De Cillis et al (2022), Italy	Single arm prospective	WJAT	60	64.9 ± 7	63.5 ± 16.8
Becker et al (2017), Germany	Single arm prospective	HoLEP	54	72.5 (67‐77.25)	74.5 (45‐110)
Gilling et al (2007)	Single arm prospective	HoLEP	71	69.1 ± 9.0	58.5 ± 31.0
Vavassori et al (2007)	Single arm prospective	HoLEP	330	66 ± 8.1	62 ± 34 cc
Abdel Hakim et al (2009)	Single arm prospective	HoLEP	230	69.8 ± 10.3	86.5 ± 65.4

Although the inclusion criteria were varied (Table S2), all trials excluded patients with kidney disease, bladder dysfunction, bladder calculi and a history of cancer. Regarding study design, among all included studies, only a single study in the HoLEP group implemented patient blinding.^33^ In terms of the longest follow‐up period and the largest sample size, WJAT studies featured a follow‐up of 60 months with 114 participants, whereas the HoLEP group demonstrated a longer follow‐up of 84 months and a larger cohort of 200 patients.^12^, ^34^, ^45^

HoLEP was commonly performed using lasers in the 80‐ to 100‐W range, whereas most WJAT studies used pre‐set computerized machines, enabling quicker procedures. Focal bladder neck cautery (FBNC) has recently been advocated with WJAT as it shows to significantly reduce bleeding.^46^ It should be noted that all studies included in this review were published before the FBNC widespread. Most RCTs on HoLEP were before 2016, and all WJAT studies were published after 2015. The baseline characteristics for most studies were similar (Tables 2A and 2B). The studies recorded various outcomes in addition to those selected for this review, such as the International Index of Erectile Function (IIEF)‐5 score, prostate‐specific antigen (PSA) and PV. Only one study commented on incidental prostate cancer (iPCa) rates for TURP and HoLEP, while no reports of iPCa were found for WJAT. Most outcomes ranged from moderate to very low QoE, with inadequate blinding in RCTs contributing to high RoB, leading to downgrading of evidence. A detailed explanation of each outcomes GRADE rating can be seen in Table S3.

### 3.2 Quantitative analysis

#### 3.2.1 IPSS (Figure 3)

**FIGURE 3:**
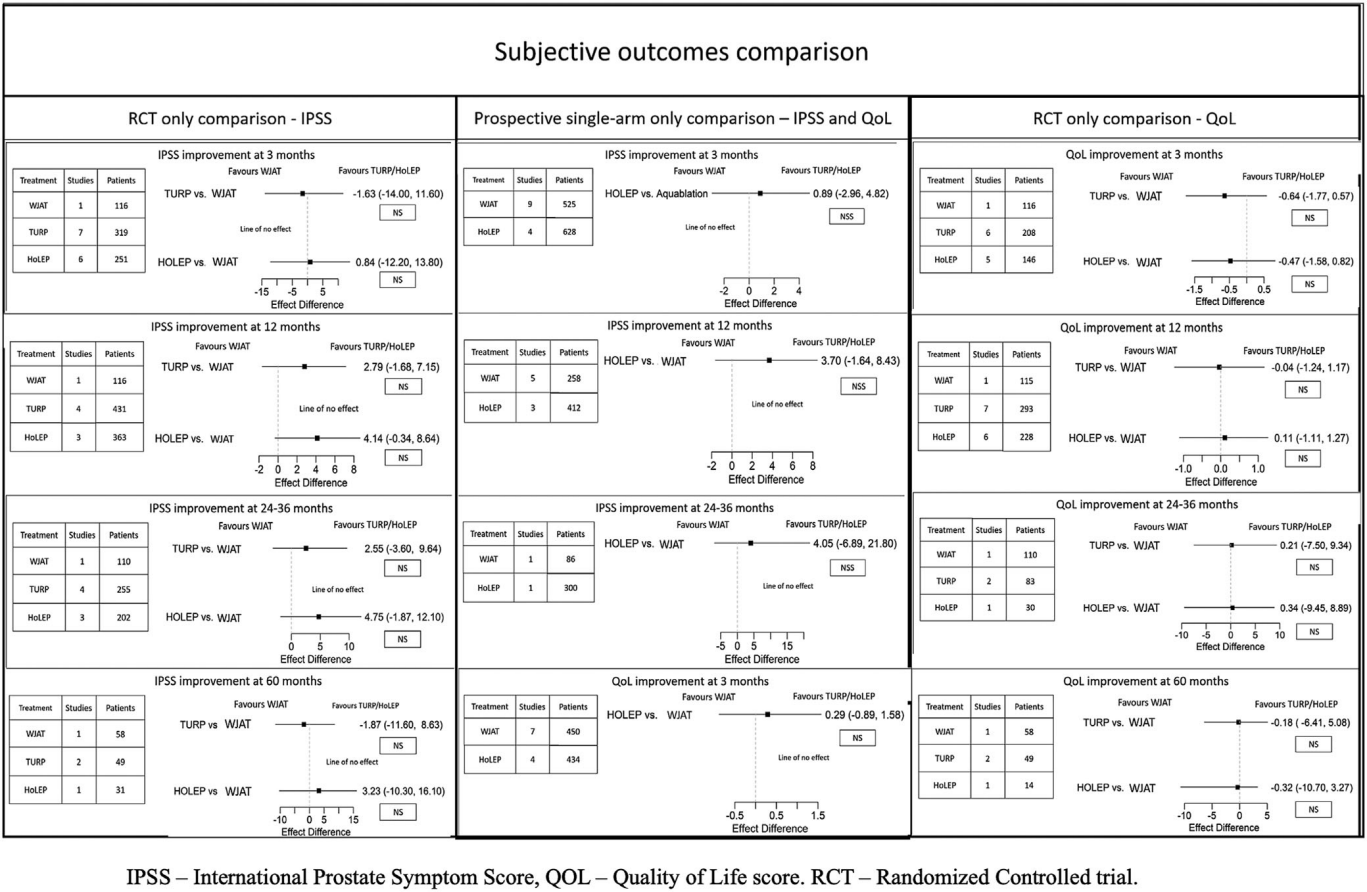
Subjective outcome comparison between WJAT and HoLEP.

In the RCT only analysis, HoLEP improved IPSS by 0.84 points (95% CI: −12.20 to 13.80, NS, GRADE rating: Low), 4.14 points (95% CI: −0.34 to 8.64, NS, GRADE rating: Low), 4.75 points (95% CI: −1.87 to 12.10, NS, GRADE rating: Low) and 3.23 points (95% CI: −10.30 to 16.10, NS, GRADE rating: Low) more compared to WJAT at 3, 12, 24–36 and 60 months, respectively. In the prospective single‐arm study analysis, HoLEP improved IPSS by 0.89 points (95% CI: −2.96 to 4.82, NS GRADE rating: Very Low), 3.70 points (95% CI: −1.64 to 8.43, NS, GRADE rating: Very Low) and 4.05 points (95% CI: −6.89 to 21.80, NS, GRADE rating: Very Low) more compared to WJAT at 3, 12 and 24–36 months.

In the RCT‐only analysis, At 3 and 60 months, WJAT improved IPSS by 1.63 points (95% CI:‐14 to 11.60, NS, GRADE rating: Low) and 1.87 points (95% CI: −11.60 to 8.63, NS, GRADE rating: Low) more compared to TURP, respectively. At 12 and 24–36 months, TURP improved IPSS by 2.79 points (95% CI: ‐1.68 to 7.15 NS GRADE rating: Low) and 2.55 points (95% CI: −3.60 to 9.64, NS, GRADE rating: Low).

#### 3.2.2 QoL score (Figure 3)

In the RCT‐only analysis, WJAT improved the QoL score by 0.47 (95% CI: −1.58 to 0.82, NS, GRADE rating: Low), 0.32 points (95% CI: −10.70 to 3.27, NS, GRADE rating: Low) more compared to HoLEP improvement at 3 and 60 months, respectively. HoLEP improved QoL score by 0.11 points (95% CI: −1.11 to 1.27, NS, GRADE rating: Low), 0.34 points (95% CI: −9.45 to 8.89, NS¸ GRADE rating: Low), more than WJAT at 12 and 24–36 months. In the prospective single‐arm study analysis, HoLEP improved QoL by 0.29 points (95% CI: −0.89 to 1.58, NS, GRADE rating: Low) more than WJAT at three months.

WJAT improved the QoL score by 0.67 (95% CI: −1.77 to 0.82, NS, GRADE rating: Low), 0.04 (95% CI: −1.24 to 1.17, NS, GRADE rating: Low) and 0.18 points (95% CI: −6.41 to 5.08, GRADE rating: Low) more than TURP at 3, 12 and 60 months, respectively. TURP improved the QoL score by 0.21 points (95% CI: −7.50 to 8.89, GRADE rating: Low) at 24–36 months.

#### 3.2.3 Qmax (Figure 4)

**FIGURE 4:**
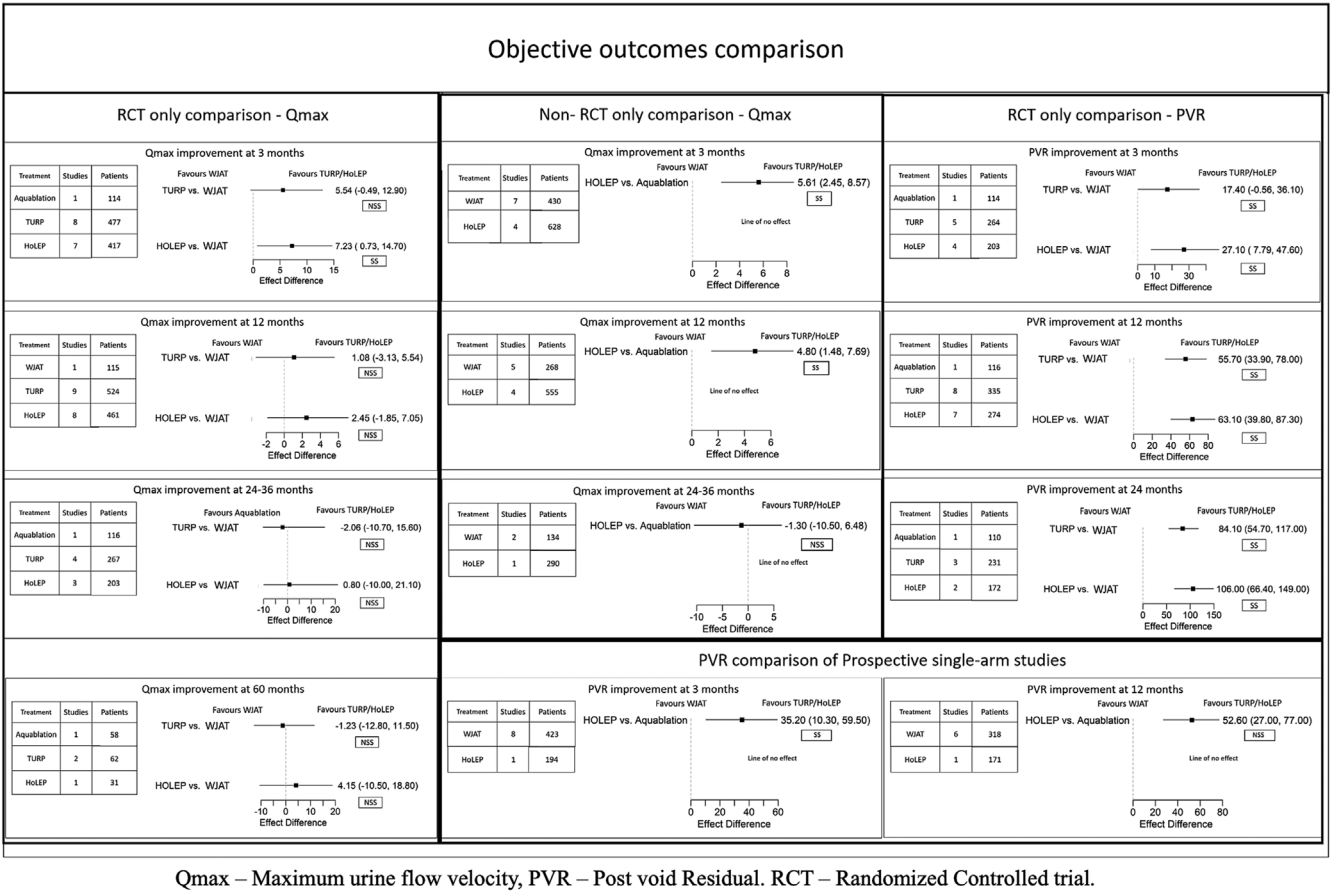
Objective outcomes comparison between WJAT and HoLEP.

In the RCT‐only analysis, HoLEP outperformed WJAT by 7.23 ml/s (95% CI: 0.73 to 14.70, SS, GRADE rating: Moderate), 2.45 (5% CI: −1.85 to 7.05, NS, GRADE rating: Low), 0.80 ml/s (95% CI: −7.72 to 12.00, NS, GRADE rating: Low) and 4.15 ml/s (95% CI: −10.50 to 18.80, NS, GRADE rating: Very Low) at 3, 12, 24–36 and 60 months, respectively. In the prospective single‐arm study analysis, HoLEP improved Qmax by 5.61 ml/s (95% CI: 2.45 to 8.57, SS, GRADE rating: Low), 4.80 ml/s (95% CI: 1.48 to 7.69, NS, GRADE rating: Low) at 3 and 12 months. WJAT improved 1.30 ml/s (95% CI: −10.50 to 6.48, NS, GRADE rating: Moderate) more than HoLEP at 24–36 months.

TURP outperformed WJAT by 5.54 ml/s (95% CI: −0.48 to 12.90, NS, GRADE rating: Moderate), 1.08 ml/s (95% CI: −3.13 to 5.54, NS, GRADE rating: Low) at 3 and 12 months. However, WJAT improved Qmax by 2.06 ml/s (95% CI: −10.70 to 15.60, GRADE rating: Low) and 1.23 ml/s (95% CI: −12.80 to 11.50, GRADE rating: Very Low) more than TURP at 24–36 and 60 months. Interestingly, while Qmax's improvement is superior to HoLEP at all other time points, improvement at 24 months is similar between the treatments in both RCT and prospective single‐arm study analysis.

#### 3.2.4 PVR (Figure 4)

In the RCT‐only analysis, HoLEP outperformed WJAT by 27.10 ml (95% CI: −7.79 to 47.60, SS, GRADE rating: Moderate), 63.10 ml (95% CI: 39.80 to 87.30, SS, GRADE rating: Moderate), 106.00 ml/s (95% CI: 66.40 to 149.00, SS, GRADE rating: Low), at 3, 12 and 24–36 months, respectively. In the prospective single‐arm study analysis, HoLEP outperformed WJAT by 35.20 (95% CI: 10.30 to 59.50, SS, GRADE rating: Low) and 52.60 (95% CI: 27.00 to 77.00, SS, GRADE rating: Low) at 3 and 12 months.

TURP outperformed WJAT by 17.40 ml (95% CI: −0.56 to 36.10, NS, GRADE rating: Moderate), 55.70 ml (95% CI: 33.90 to 78.00, NS, GRADE rating: Moderate), 84.10 (95% CI: 55.70 to 117.00, NS, GRADE rating: Low), at 3, 12 and 24–36 months, respectively.

#### 3.2.5 Erectile function (Figure S1)

HoLEP and WJAT were almost identical in the RCT‐only analysis, with WJAT causing an increase of 0.02 (95% CI: −4.42 to 7.67, NS, GRADE rating: Low) compared to HoLEP for IIEF‐score improvement at three months. Similarly, at 12 months, HoLEP resulted in an IIEF‐score increase of 0.13 points (95% CI: −6.21 to 8.85, GRADE rating: Low) compared to WJAT. Further time points could not be evaluated due to a lack of studies.

#### 3.2.6 Perioperative outcomes (Figure 5)

**FIGURE 5:**
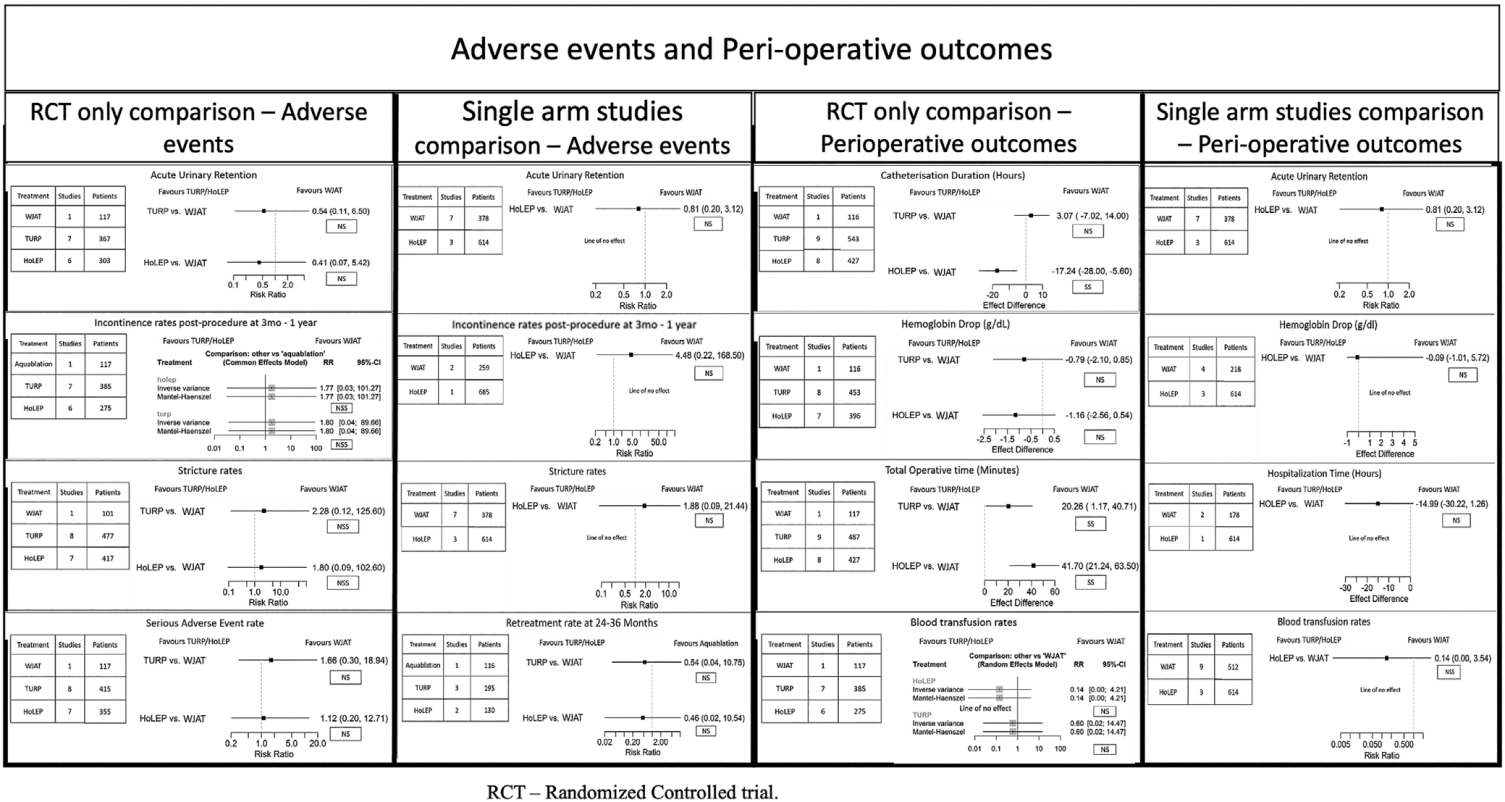
Adverse event comparison and perioperative outcomes between WJAT and HoLEP. RCT – randomized controlled trial.

##### Acute urinary retention

In the RCT‐only analysis, HoLEP had a lower risk ratio of AUR (RR = 0.41, 95% CI: 0.07 to 5.42, NS, GRADE rating: Very Low) compared to WJAT. While the prospective single‐arm study analysis had a slightly higher rate of AUR with HoLEP (RR = 0.81, 95% CI: 0.20 to 3.12, NS, GRADE rating: Very Low), the overall trend is one of HoLEP having lower AUR rates, though NS.

##### Catheterization duration

In the RCT‐only analysis, HoLEP had a lower catheterization duration of 17.24 hours (95% CI: −28.00 to −5.00 hours, SS, GRADE rating: Moderate) than WJAT. This is in line with the prospective single‐arm study analysis, showing a significantly shorter duration of catheterization with HoLEP.

##### Postoperative Haemoglobin drop

In the RCT‐only analysis, HoLEP was associated with a lower haemoglobin (Hb) loss of 1.16 mg/dl (95% CI: −2.56 to 0.54 mg/dl, NS, GRADE rating: Moderate) compared to WJAT. This result disagrees with the prospective single‐arm study analysis, which shows only a difference of 0.09 mg/dl. (95% CI: −1.01 to 5.72, NS, GRADE rating: Low).

##### Total operating time

In the RCT‐only analysis, the resection time was 41.70 minutes (95% CI: 21.24 to 63.50, SS, GRADE rating: Moderate) higher with HoLEP than WJAT. The prospective single‐arm study analysis also showed a significantly higher resection time with HoLEP, albeit only 11.65 (95% CI: 0.35 to 23.81, SS, GRADE rating: Low) minutes longer.

##### Hospitalization time

In the RCT‐only analysis, hospitalization time was 3.26 hours (95% CI: −20.88 to 14.78, SS, GRADE rating: Low), higher with HoLEP than WJAT. This result disagrees with the prospective single‐arm study analysis, which shows a 15‐hour shorter duration of hospitalization with HoLEP compared to WJAT, though the result did not reach significance (GRADE rating: Low).

#### 3.2.7 Complications (Figure 5)

##### Incontinence

In the RCT‐only analysis, there was a minimal difference in the risk of incontinence post‐procedure (RR = 1.09, higher with HoLEP, 95% CI: 0.09 to 26.42, NS, GRADE rating: Very Low) with HoLEP compared to WJAT. The prospective single‐arm study analysis indicated that HoLEP had a much higher RR of 4.48 (95% CI: 0.22 to 168.50, NS, GRADE rating: Very Low) for incontinence compared to WJAT.

##### Urethral stricture

In the RCT‐only analysis, there was a higher risk of urethral stricture (RR = 1.80, higher with HoLEP, 95% CI: 0.09 to 102.60, NS, GRADE rating: Very Low) with HoLEP compared to WJAT. This result is in line with the prospective single‐arm study analysis (RR = 1.88, higher with HoLEP, 95% CI: 0.09 to 21.40, NS, GRADE rating: Very Low).

##### Blood transfusion

In the RCT‐only analysis, there was a higher risk of blood transfusion (RR = 0.14, higher with WJAT, 95% CI: 0.00 to 4.21, NS, GRADE rating: Low) with WJAT compared to HoLEP. This result is in line with the prospective single‐arm study analysis (RR: 0.14, 95% CI: 0.0 to 3.54, in favour of HoLEP, NSS, GRADE rating: Low).

##### Total serious adverse events (SAE)

In the RCT‐only analysis, there was a higher risk of SAE post‐procedure (RR = 1.12, higher with HoLEP, 95% CI: 0.20 to 12.71, NS, GRADE rating: Low) with HoLEP compared to WJAT.

##### Retreatment

In the RCT‐only analysis, RTR was lower with HoLEP (RR = 0.46, 95% CI: 0.02 to 10.54, GRADE rating: Low). The same outcome was not evaluated in prospective single‐arm studies due to inadequate data.

**FIGURE 6:**
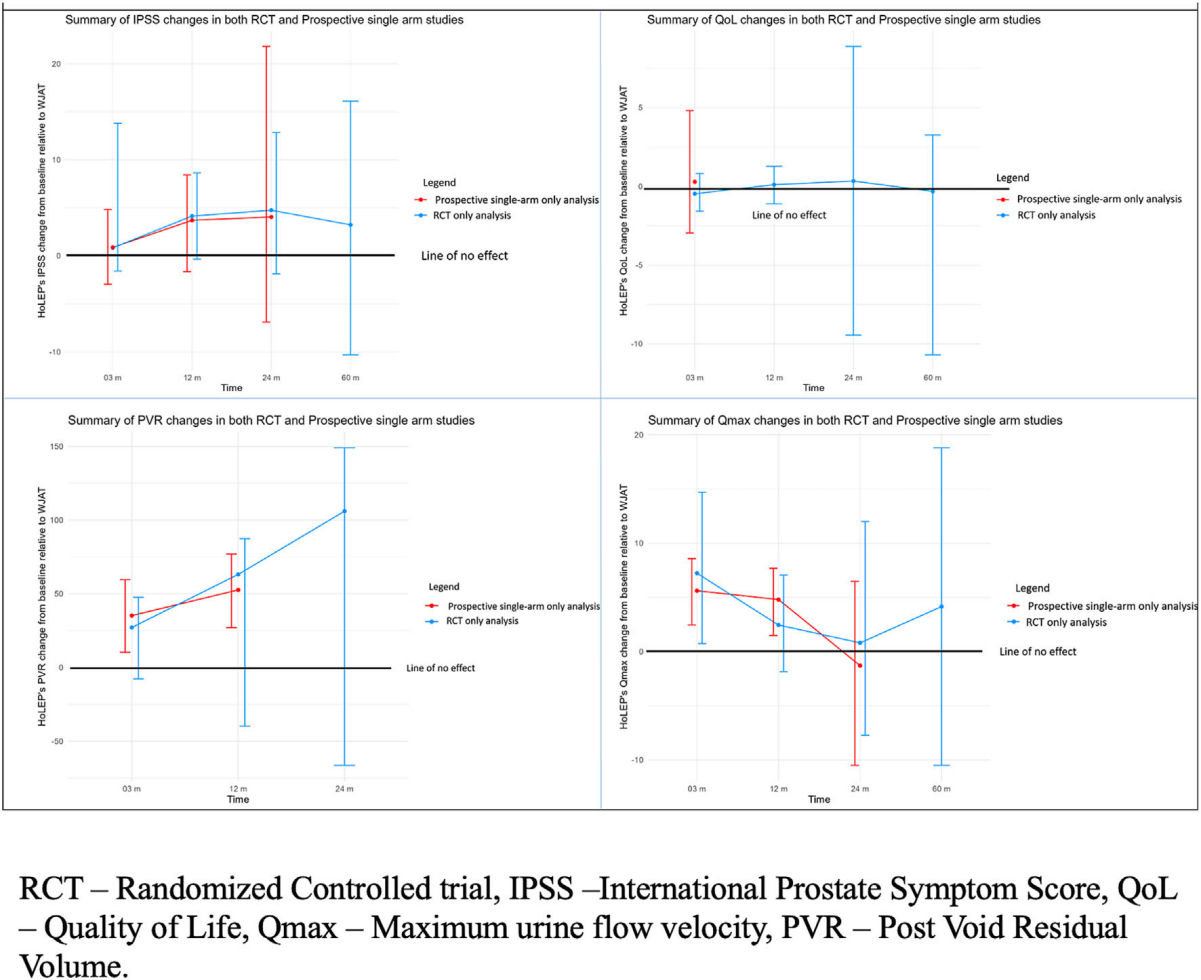
Summary figure of major efficacy outcomes between WJAT and HoLEP. RCT – randomized controlled trial, IPSS –international prostate symptom score, QoL – quality of life, Qmax – maximum urine flow velocity, PVR – post void residual volume.

Figure 6 shows a summary of the primary functional outcomes. Although statistically insignificant, WJAT has approximately 4‐point lower IPSS improvement than HoLEP at 1, 2–3 and 5‐year postoperative periods (Figure 6). A 3–4 point difference in IPSS is considered the Minimal Clinically Important Difference (MCID).^47^, ^48^, ^49^

## 4 DISCUSSION

The dynamic field of BPH treatment has recognized HoLEP as a size‐independent procedure with satisfactory outcomes, albeit challenged by a steep learning curve.^50^ In contrast, WJAT, currently exemplified by Aquablation, has been developed with promises of similar satisfactory outcomes, with an added advantage of potentially preserving ejaculation and a more manageable learning curve due to the incorporation of robotic assistance. Our study revealed that HoLEP outperforms WJAT in Qmax up to one year and PVR up to five years post‐procedure. Additionally, HoLEP showed more favourable outcomes in postoperative catheterization duration. Conversely, WJAT outperformed HoLEP in operative time and hospital stay length post‐procedure. Despite identifying advantages and disadvantages for each procedure, our analysis suggests that WJAT and HoLEP are valuable additions to an urologist's armamentarium, necessitating careful evaluation to determine the best scenarios for their application.

Although borderline statistically insignificant, WJAT has approximately 4‐point lower IPSS improvement than HoLEP at 1, 2–3 and 5‐year postoperative periods (Figure 6). In line with our result, HoLEP has been shown to have the highest IPSS reduction out of all transurethral procedures, and WJAT has previously been shown to have similar IPSS improvement to TURP.^51^, ^52^ Despite differences in IPSS, we found that WJAT has a similar improvement in QoL to HoLEP and TURP, indicating that most patients are equally satisfied after each procedure. This trend was noted at all time points.

HoLEP outperformed WJAT in Qmax improvement on analysing RCTs (especially at three‐month and five‐year follow‐up periods). However, the difference was not significant at later time‐points, likely due to smaller sample sizes. Contrarily to this finding, prospective studies' analysis revealed significant superior Qmax improvement at three‐month and one‐year follow‐ups with HoLEP. The divergence in outcomes may be attributed to the data from the WATER trial (the only RCT reporting the Qmax at these time points), as WJAT performed closely to TURP at these time points. Moreover, we also noted that PVR improvement was consistently better with HoLEP. Enucleation was not only significantly superior at all follow‐up time frames, but there was also an increase in PVR improvement from 27.1 ml at three months to 63.1 ml at one year and 108 ml at two years. Although this finding can be due to the higher baseline residual volumes in two HoLEP studies,^34^, ^42^ the overall data indicates that PVR improvement is significantly better with HoLEP than with WJAT.^8^, ^34^, ^36^, ^42^

The better performance of HoLEP in objective outcomes can be explained by the almost complete adenomectomy achieved by HoLEP, which results in an almost 90% reduction in the adenoma size.^53^, ^54^ The drop in PSA after endoscopic enucleation is reported to be between 75% and 90% with a nadir level between 0.5 and 1 ng/dl.^55^ On the other hand, the mean drop in PSA was 27% (2.7 ± 2.3 ng/ml) of the baseline value for WATER‐I and 38% (4.4 ± 4.3 ng/ml) of the baseline value for the WATER‐II study.^56^ This reduction in PSA is significantly lesser than enucleation but is comparable to other resection techniques like TURP around 12 months after the procedure, which is 30–40% of the baseline.^55^

The data regarding volume reduction post WJAT is sparse, with only two single‐arm studies reporting 3‐month PV measurements,^23^, ^32^ which reported a mean change of − 46.48 g and −20 g, respectively. Considering PSA as a surrogate marker for PV, it can be extrapolated that WJAT might leave behind more residual adenoma, limiting objective voiding improvement evaluated by non‐invasive urodynamic parameters of Qmax and PVR. The only study reporting PV change after HoLEP reported a decrease from 77.8 ± 5.6 g to 28.4 ± 1.8 g at six months.

An important topic to be discussed with patients is the association of an enlarged prostate with sexual outcomes. It is well‐known that BPH symptoms are associated with erectile dysfunction. Multiple studies have been conducted to quantify the impact of surgical procedures for BPH on male potency.^57^ In the present study, we found that erectile function was not significantly impacted with either procedure at three months or one year. Previous studies demonstrated that TURP, HoLEP and Aquablation do not affect erection.^58^, ^59^

However, beyond the erectile function, the ability to preserve ejaculation also represents an important topic for men.^60^ While the incidence of retrograde ejaculation with endoscopic enucleation of the prostate (EEP) is around 70%,^61^ The major advantage of WJAT is its ability to preserve antegrade ejaculation with an anejaculation rate of 10% and 15%, as noted in both WATER I and II studies.^12^, ^56^ Although one can argue that some patients may not be concerned about losing antegrade ejaculation, this might still be an essential aspect of sexual satisfaction in other men.^60^ This is attributed to its template wherein the tissue 1 cm proximal to verumontanum is preserved, sparing the *para‐collicular tissue*.^62^ Patients undergoing WJAT would have to balance preserving ejaculation by means of incomplete resection of the adenoma with the lower efficacy in the LUTS treatment mentioned above. There is a recent rise in studies focusing on modified techniques focusing on the preservation of antegrade ejaculation in HoLEP and TURP.^63^, ^64^, ^65^ In the future, directly comparing the outcome of the ejaculation‐preserving template for EEP with WJAT would benefit patient selection.

WJAT is a robotic procedure aiming to improve ergonomics and reduce operation time.^66^ Our study revealed that WJAT has a significantly lower total operation time of 41 minutes compared to HoLEP. However, this was associated with significantly higher postoperative catheter duration. Additionally, length of stay, urine retention and haemoglobin drop were higher with WJAT, although the difference was insignificant. We noted that the haemoglobin drop postoperatively is about 1.2 mg/dl higher with WJAT than with HoLEP. This increased haemoglobin loss is similar to that seen with TURP.^5^ The higher rate of bleeding and blood transfusion requirement with WJAT might be explained by initial sub‐optimal haemostasis techniques employed for WJAT.^46^ However, after FBNC introduction, there is a significant reduction in these complications, which has not been accounted for in the included studies.^46^ However, the introduction of FBNC is also likely to increase the operative time for WJAT, possibly decreasing the time difference in both procedures.

Adverse events were found to be higher with HoLEP than WJAT in the NMA of the included studies. However, real‐world adverse events with WJAT have been extensively reported in the MAUDE and other adverse event reporting databases. According to two reviews comparing WJAT to other operative techniques for BPH, WJAT had significantly higher adverse events.^67^, ^68^ One review found that haematuria, rectal perforation, clot retention and prostate capsule perforation were associated with WJAT.^68^ Database studies have not reported complications with HoLEP outside of those reported in the studies included in this NMA.

This analysis found that the incontinence rate at up to 12 months postoperatively was similar between both procedures in the RCT‐only analysis but was almost five times higher with HoLEP in the prospective single‐arm study analysis. The single‐arm comparison analysis had multiple studies with 0 incontinence events in the WJAT group, while the WATER trial (Part of the RCT‐only analysis) did not adequately report their incontinence findings, explaining the differences. We believe that in this scenario, the prospective single‐arm analysis is likely to represent real‐world effects more closely, and the RCT‐only analysis is due to a quirk of the statistical modelling due to zero events and unclear reporting.

HoLEP is considered a size‐independent procedure for the surgical management of BPH.^3^ Similarly, the WATER 2 trial showed WJAT's viability with prostates > 80 g despite the higher bleeding and incontinence rates associated with larger prostates.^31^ A recent study comparing the HoLEP outcome in different prostate sizes revealed a higher need for blood transfusion for prostates > 200 g.^9^ These results indicate that HoLEP and WJAT are likely viable options for larger prostates, with operator experience and patient preference being the determining factors between such cases. The SAE rate was almost similar between both procedures and was slightly higher with TURP than with WJAT. Given HoLEP's and WJAT's less‐invasive nature, their SAE rates are expected to be lower than TURP's. Other reviews have found that HoLEP has a lower SAE rate than TURP.^8^ Similarly, WJAT has been shown to have a lower rate SAE rate than TURP.^69^

None of the studies on WJAT provide information about iPCa detection. This is likely due to insufficient quality tissue samples for postoperative histopathological evaluation (HPE).^70^ The lack of iPCa detection may lead to a small but significant percentage of prostate cancer patients being missed.

Most comparisons in this context receive a GRADE rating of Moderate,’ with a few rated as ‘Low or ‘Very Low.’ All RCT comparisons were downgraded at least one level due to the RoB in some studies which originated from inadequate blinding of participants in all RCTs except one. However, in RCTs comparing surgical outcomes, achieving blinding is frequently challenging. In summary, we believe that while there is a clear RoB with such study designs, it is difficult to remove it entirely. The results presented in this NMA indicate that WJAT is an effective procedure that can provide similar results to HoLEP in subjective outcomes, and it may serve as a size‐independent treatment with lower AE rates than HoLEP. However, it results in a lower PSA reduction and may not provide adequate tissue for postoperative HPE.

## 5 LIMITATIONS AND FUTURE RESULTS

This study has several limitations. We included only 536 patients in the WJAT cohort. Since most RCTs comparing HoLEP and TURP were published before 2010, improvements in HoLEP technology/technique may not be represented. The different institutional protocols used in WJAT procedures, different inclusion/exclusion criteria for both WJAT and HoLEP studies, different laser devices, different power settings and energy sources used and differential operator preferences can limit the generalizability of the findings. Moreover, an additional consideration in BPH treatment is its impact on ejaculatory function, which could not be assessed in the current study due to the fact that none of the RCTs for HoLEP included measures of ejaculatory function. We did not perform a sub‐group analysis as both treatments are considered size‐independent.

Although WJAT takes less operative time than HoLEP, the need for focal bladder neck cauterization and resection of fluffy/residual tissue adds complexity to the procedure with its additional time. Since most of the studies included in the present NMA did not resort to FNBC and TUR, the difference in operating time between HoLEP and WJAT found in our NMA may not be reflective of current practice. We also did not compare the prostate volume and PSA reduction given the scarce data in WJAT studies. Given the lack of long‐term data on WJAT other than the WATER trial, the RTR and long‐term comparative efficacy of WJAT remain an open question.

Nonetheless, we provide the first study to date comparing these two popular techniques. Our findings suggest that although HoLEP provides better outcomes regarding Qmax and PVR improvement, the shorter resection time of WJAT may represent a faster turnover time and improved economic incentives, especially for outpatient centres. Future research may focus on trials with longer follow‐ups to directly compare the effectiveness and safety of WJAT, HoLEP and TURP; a few such trials are underway.^71^ These studies should also provide data on the differences between these procedures related to prostate volume and PSA reduction. Standardized protocols implemented for multi‐centric trials for all three procedures may help reduce heterogeneity and reveal a more uniform effect size. These studies will capture data of HoLEP with updated technical modification and WJAT after implementing FNBC as the standard approach.

## 6 CONCLUSION

HoLEP demonstrates superior performance in Qmax and PVR compared to WJAT, though WJAT exhibits non‐inferiority in subjective outcomes, such as IPSS, QoL and SHIM/IIEF‐5 and parallels HoLEP in terms of erectile function results. WJAT is associated with a shorter resection time, but a higher haemoglobin drop. It also presents lower incontinence rates, while maintaining comparable rates of SAEs and urethral strictures in relation to HoLEP.

## AUTHOR CONTRIBUTIONS

**Ansh Bhatia:** Conceptualization; data curation; formal analysis; investigation; methodology; software; validation; visualization; writing—original draft preparation; writing—review and editing. **Renil S. Titus:** Data curation; formal analysis; investigation; methodology; software; visualization; writing—review and editing. **Joao G. Porto:** Data curation; formal analysis; investigation; methodology; project administration; validation; visualization; writing—review and editing. **Rajvi Goradia:** Data curation; validation; visualization. **Khushi Shah:** Data curation; validation; visualization; writing—review and editing. **Diana Lopategui:** Project administration; supervision; validation; writing—review and editing. **Thomas R.W. Herrmann:** Conceptualization; investigation; methodology; project administration; supervision; visualization; writing—review and editing. **Hemendra N. Shah:** Conceptualization; formal analysis; investigation; methodology; project administration; supervision; validation; visualization; writing—original draft preparation; writing—review and editing.

## CONFLICT OF INTEREST STATEMENT

All authors declare that they have no conflict of interests.

## Supporting information


**Table S1.** Outcomes of sensitivity analysis.
**Table S2.** Inclusion and Exclusion criteria of studies.
**Table S3.** GRADE ratings.
**Figure S1.** Supplementary: IIEF‐5 outcomes comparing EF change at 3 and 12 months.

## References

[1] Launer BM , McVary KT , Ricke WA , Lloyd GL . The rising worldwide impact of benign prostatic hyperplasia. BJU Int. 2021;127(6):722–728. 10.1111/bju.15286 33124118 PMC8170717

[2] Awedew AF , Han H , Abbasi B , Abbasi‐Kangevari M , Ahmed MB , Almidani O , et al. The global, regional, and national burden of benign prostatic hyperplasia in 204 countries and territories from 2000 to 2019: a systematic analysis for the global burden of disease study 2019. Lancet Health Longev. 2022;3(11):e754–e776. 10.1016/S2666-7568(22)00213-6 PMC9640930 36273485

[3] Lerner LB , McVary KT , Barry MJ , Bixler BR , Dahm P , Das AK , et al. Management of lower urinary tract symptoms attributed to benign prostatic hyperplasia: AUA GUIDELINE PART I—initial work‐up and medical management. J Urol. 2021;206(4):806–817. 10.1097/JU.0000000000002183 34384237

[4] Pujari N . Transurethral resection of prostate is still the gold standard for small to moderate sized prostates. J Integr Nephrol Androl. 2016;3(2):68. 10.4103/2394-2916.181223

[5] Rassweiler J , Teber D , Kuntz R , Hofmann R . Complications of transurethral resection of the prostate (TURP)—incidence, management, and prevention. Eur Urol. 2006;50(5):969–980. 10.1016/j.eururo.2005.12.042 16469429

[6] Banks F . HoLEP: the new ‘gold standard’ in bladder outflow surgery. Trends Urol Men's Health. 2017;8(3):26–28. 10.1002/tre.582

[7] Jones P , Alzweri L , Rai BP , Somani BK , Bates C , Aboumarzouk OM . Holmium laser enucleation versus simple prostatectomy for treating large prostates: results of a systematic review and meta‐analysis. Arab J Urol. 2016;14(1):50–58. 10.1016/j.aju.2015.10.001 26966594 PMC4767783

[8] Chen J , Dong W , Gao X , Li X , Cheng Z , Hai B , et al. A systematic review and meta‐analysis of efficacy and safety comparing holmium laser enucleation of the prostate with transurethral resection of the prostate for patients with prostate volume less than 100 mL or 100 g. Transl Androl Urol. 2022;11(4):407–420. 10.21037/tau-21-1005 35558272 PMC9085931

[9] Porto JG , Blachman‐Braun R , Delgado C , Zarli M , Chen R , Ajami T , et al. Is holmium laser enucleation of the prostate truly size‐independent? A critical evaluation at the extreme ends of the Spectrum. Urology. 2023;1(182):204–210. 10.1016/j.urology.2023.09.002 37716456

[10] Zhang TR , Thorogood SL , Sze C , Fisch R , Chughtai B , Te A , et al. Current practice patterns in the surgical management of benign prostatic hyperplasia. Urology. 2023;1(175):157–162. 10.1016/j.urology.2023.02.025 36863599

[11] McVary KT , Gittelman MC , Goldberg KA , Patel K , Shore ND , Levin RM , et al. Final 5‐year outcomes of the multicenter randomized sham‐controlled trial of a Water vapor thermal therapy for treatment of moderate to severe lower urinary tract symptoms secondary to benign prostatic hyperplasia. J Urol. 2021;206(3):715–724. 10.1097/JU.0000000000001778 33872051

[12] Gilling P , Barber N , Bidair M , Anderson P , Sutton M , Aho T , et al. WATER: a double‐blind, randomized, controlled trial of Aquablation® vs transurethral resection of the prostate in benign prostatic hyperplasia. J Urol. 2018;199(5):1252–1261. 10.1016/j.juro.2017.12.065 29360529

[13] Sandhu JS , Bixler BR , Dahm P , Goueli R , Kirkby E , Stoffel JT , et al. Management of lower urinary tract symptoms Attributed to benign prostatic hyperplasia (BPH): AUA guideline amendment 2023. J Urol. 2024;211(1):11–19. 10.1097/JU.0000000000003698 37706750

[14] Uroweb ‐ European Association of Urology . [Internet]. [cited 2023 Feb 4]. EAU guidelines on the management of non‐neurogenic male LUTS ‐ INTRODUCTION ‐ Uroweb. Available from: https://uroweb.org/guidelines/management-of-non-neurogenic-male-luts/chapter/introduction

[15] UPDATE – 2022 Canadian Urological Association guideline on male lower urinary tract symptoms/benign prostatic hyperplasia (MLUTS/BPH)|Canadian Urological Association Journal [Internet]. [cited 2024 Jan 21]. Available from: https://cuaj.ca/index.php/journal/article/view/7906

[16] Omidele OO , Siegal AS , Roshandel R , Te AE , Kaplan SA . Aquablation at 4‐years: real world data from the largest single‐center study with associated outcomes follow‐up. Urology. 2024. 10.1016/j.urology.2024.07.047 39084348

[17] Study Quality Assessment Tools|NHLBI, NIH [Internet]. [cited 2022 May 16]. Available from: https://www.nhlbi.nih.gov/health-topics/study-quality-assessment-tools

[18] Izcovich A , Chu DK , Mustafa RA , Guyatt G , Brignardello‐Petersen R . A guide and pragmatic considerations for applying GRADE to network meta‐analysis. BMJ. 2023;27(381):e074495. 10.1136/bmj-2022-074495 37369385

[19] Pergialiotis V , Bellos I , Biliou EC , Varnava P , Mitsopoulou D , Doumouchtsis SK , et al. An arm‐based network meta‐analysis on treatments for vulvar lichen sclerosus and a call for development of core outcome sets. Am J Obstet Gynecol. 2020;222(6):542–550.e6. 10.1016/j.ajog.2019.10.095 31697910

[20] Aoyama H , Uchida K , Aoyama K , Pechlivanoglou P , Englesakis M , Yamada Y , et al. Assessment of therapeutic interventions and lung protective ventilation in patients with moderate to severe acute respiratory distress syndrome: a systematic review and network meta‐analysis. JAMA Netw Open. 2019;2(7):e198116. 10.1001/jamanetworkopen.2019.8116 31365111 PMC6669780

[21] Bhatia A , Porto JG , Maini A , Langade D , Herrmann TRW , Shah HN , et al. One‐year outcomes after prostate artery embolization versus laser enucleation: a network meta‐analysis. BJUI Compass. 2024;5(2):189–206. 10.1002/bco2.302 38371212 PMC10869668

[22] Bhatia A , Porto JG , Titus RS , Ila V , Shah K , Malpani A , et al. A systematic review and network meta‐analysis comparing Rezūm with transurethral needle ablation and microwave thermotherapy for the management of enlarged prostate. BJUI Compass. 2024;5(7):621–635. 10.1002/bco2.361 39022654 PMC11250421

[23] Bach T , Giannakis I , Bachmann A , Fiori C , Gomez‐Sancha F , Herrmann TRW , et al. Aquablation of the prostate: single‐center results of a non‐selected, consecutive patient cohort. World J Urol. 2019;37(7):1369–1375. 10.1007/s00345-018-2509-y 30288598

[24] Desai MM , Singh A , Abhishek S , Laddha A , Pandya H , Ashrafi AN , et al. Aquablation therapy for symptomatic benign prostatic hyperplasia: a single‐Centre experience in 47 patients. BJU Int. 2018;121(6):945–951. 10.1111/bju.14126 29319914

[25] Yee CH , Tang SF , Yuen SKK , Chan CK , Teoh JYC , Chiu PKF , et al. Technique, outcome and changes in prostate dimensions in patients with urinary retention managed by aquablation. Int Urol Nephrol. 2022;54(8):1787–1792. 10.1007/s11255-022-03244-y 35622268 PMC9136199

[26] Helfand BT , Glaser AP , Kasraeian A , Sterious S , Talaty P , Alcantara M , et al. Men with lower urinary tract symptoms secondary to BPH undergoing Aquablation with very large prostates (> 150 mL). Can J Urol. 2021;28(6):10884–10888. 34895392

[27] Labban M , Mansour M , Abdallah N , Jaafar R , Wazzan W , Bulbul M , et al. Aquablation for benign prostatic obstruction: single center technique evolution and experience. Invest Clin Urol. 2021;62(2):210–216. 10.4111/icu.20200249 PMC7940846 33660449

[28] Misrai V , Rijo E , Zorn KC , Barry‐Delongchamps N , Descazeaud A . Waterjet ablation therapy for treating benign prostatic obstruction in patients with small‐ to medium‐size glands: 12‐month results of the first French Aquablation clinical registry. Eur Urol. 2019;76(5):667–675. 10.1016/j.eururo.2019.06.024 31281024

[29] Whiting D , Ng KL , Barber N . Initial single centre experience of Aquablation of the prostate using the AquaBeam system with athermal haemostasis for the treatment of benign prostatic hyperplasia: 1‐year outcomes. World J Urol. 2021;39(8):3019–3024. 10.1007/s00345-020-03534-z 33392647

[30] De Cillis S , Amparore D , Quarà A , Checcucci E , Piana A , Volpi G , et al. Evaluation of LUTS of the filling phase after Aquablation: a prospective single center experience. Front Urol. 2022;2:1001710. 10.3389/fruro.2022.1001710

[31] Desai M , Bidair M , Bhojani N , Trainer A , Arther A , Kramolowsky E , et al. WATER II (80–150 mL) procedural outcomes. BJU Int. 2019;123(1):106–112. 10.1111/bju.14360 29694702

[32] Gilling P , Anderson P , Tan A . Aquablation of the prostate for symptomatic benign prostatic hyperplasia: 1‐year results. J Urol. 2017;197(6):1565–1572. 10.1016/j.juro.2017.01.056 28111300

[33] Sun N , Fu Y , Tian T , Gao J , Wang Y , Wang S , et al. Holmium laser enucleation of the prostate versus transurethral resection of the prostate: a randomized clinical trial. Int Urol Nephrol. 2014;46(7):1277–1282. 10.1007/s11255-014-0646-9 24492988

[34] Kuntz RM , Lehrich K , Ahyai S . Transurethral holmium laser enucleation of the prostate compared with transvesical open prostatectomy: 18‐month follow‐up of a randomized trial. J Endourol. 2004;18(2):189–191. 10.1089/089277904322959851 15072629

[35] Becker B , Gross AJ , Netsch C . Safety and efficacy using a low‐powered holmium laser for enucleation of the prostate (HoLEP): 12‐month results from a prospective low‐power HoLEP series. World J Urol. 2018;36(3):441–447. 10.1007/s00345-017-2159-5 29275506

[36] Gupta N , Sivaramakrishna KR , Dogra PN , Seth A . Comparison of standard transurethral resection, transurethral vapour resection and holmium laser enucleation of the prostate for managing benign prostatic hyperplasia of >40 g. BJU Int. 2006;97(1):85–89. 10.1111/j.1464-410X.2006.05862.x 16336334

[37] Wilson LC , Gilling PJ , Williams A , Kennett KM , Frampton CM , Westenberg AM , et al. A randomised trial comparing holmium laser enucleation versus transurethral resection in the treatment of prostates larger than 40grams: results at 2 years. Eur Urol. 2006;50(3):569–573. 10.1016/j.eururo.2006.04.002 16704894

[38] Montorsi F , Naspro R , Salonia A , Suardi N , Briganti A , Zanoni M , et al. Holmium laser enucleation versus transurethral resection of the prostate: results from a 2‐center, prospective, randomized trial in patients with obstructive benign prostatic hyperplasia. J Urol. 2004;172(5):1926–1929. 10.1097/01.ju.0000140501.68841.a1 15540757

[39] Bašić D , Stanković J , Potić M , Ignjatović I , Stojković I . Holmium laser enucleation versus transurethral resection of the prostate: a comparison of clinical results. Acta Chir Iugosl. 2013;60(1):15–20. 10.2298/ACI1301015B 24669558

[40] Abdel‐Hakim AM , Habib EI , El‐Feel AS , Elbaz AG , Fayad AM , Abdel‐Hakim MA , et al. Holmium laser enucleation of the prostate: initial report of the first 230 Egyptian cases performed in a single center. Urology. 2010;76(2):448–452. 10.1016/j.urology.2009.12.035 20223507

[41] Fayad AS , Sheikh MGE , Zakaria T , Elfottoh HA , Alsergany R . Holmium laser enucleation versus bipolar resection of the prostate: a prospective randomized study. Which to choose? J Endourol. 2011;25(8):1347–1352. 10.1089/end.2011.0059 21745115

[42] Jhanwar A , Sinha RJ , Bansal A , Prakash G , Singh K , Singh V . Outcomes of transurethral resection and holmium laser enucleation in more than 60 g of prostate: a prospective randomized study. Urol Ann. 2017;9(1):45–50. 10.4103/0974-7796.198904 28216929 PMC5308038

[43] Vavassori I , Valenti S , Naspro R , Vismara A , Dell'Acqua V , Manzetti A , et al. Three‐year outcome following holmium laser enucleation of the prostate combined with mechanical morcellation in 330 consecutive patients. Eur Urol. 2008;53(3):599–604. 10.1016/j.eururo.2007.10.059 17997021

[44] Gilling PJ , Aho TF , Frampton CM , King CJ , Fraundorfer MR . Holmium laser enucleation of the prostate: results at 6 years. Eur Urol. 2008;53(4):744–749. 10.1016/j.eururo.2007.04.052 17475395

[45] Gilling PJ , Wilson LC , King CJ , Westenberg AM , Frampton CM , Fraundorfer MR . Long‐term results of a randomized trial comparing holmium laser enucleation of the prostate and transurethral resection of the prostate: results at 7 years. BJU Int. 2012;109(3):408–411. 10.1111/j.1464-410X.2011.10359.x 21883820

[46] Elterman DS , Foller S , Ubrig B , Kugler A , Misrai V , Porreca A , et al. Focal bladder neck cautery associated with low rate of post‐Aquablation bleeding. Can J Urol. 2021;28(2):10610–10613. 33872559

[47] Sapoval M , Thiounn N , Descazeaud A , Déan C , Ruffion A , Pagnoux G , et al. Prostatic artery embolisation versus medical treatment in patients with benign prostatic hyperplasia (PARTEM): a randomised, multicentre, open‐label, phase 3, superiority trial. Lancet Reg Health – Eur. 2023;31. Available from:. PMID: https://www.thelancet.com/journals/lanepe/article/PIIS2666-7762(23)00091-1/fulltext 10.1016/j.lanepe.2023.100672 PMC10320610 37415648

[48] McVary KT , Miller LE , Bhattacharyya S , DeRouen K , Turner E , Zantek P , et al. Water vapor thermal therapy in men with prostate volume ≥80 cm^3^: a systematic review and meta‐analysis. Urology. 2023 Available from:. PMID: https://www.sciencedirect.com/science/article/pii/S0090429523010233 10.1016/j.urology.2023.10.036 38006957

[49] Jung JH , Reddy B , McCutcheon KA , Borofsky M , Narayan V , Kim MH , et al. Prostatic urethral lift for the treatment of lower urinary tract symptoms in men with benign prostatic hyperplasia. Cochrane Database Syst Rev. 2019;2019(5):CD012832. 10.1002/14651858.CD012832.pub2 PMC6535104 31128077

[50] Luu T , Gonzalez RR . Residency surgical BPH training paradigms from MIST to HOLEP. Curr Urol Rep. 2023;24(6):261–269. 10.1007/s11934-023-01153-w 36947390

[51] Sun F , Yao H , Bao X , Wang X , Wang D , Zhang D , et al. The efficacy and safety of HoLEP for benign prostatic hyperplasia with large volume: a systematic review and meta‐analysis. Am J Mens Health. 2022;16(4):15579883221113203. 10.1177/15579883221113203 35864746 PMC9310232

[52] Chen DC , Qu L , Webb H , Qin K , Chislett B , Xue A , et al. Aquablation in men with benign prostate hyperplasia: a systematic review and meta‐analysis. Curr Urol. 2023;17(1):68. 10.1097/CU9.0000000000000122 37692142 PMC10487298

[53] Bhat A , Katz JE , Acharya VK , Shah K , Blachman Braun R , Anthony Smith N , et al. Morphometric analysis of prostate zonal anatomy after transurethral resection of prostate and holmium laser enucleation of prostate using magnetic resonance imaging: a pilot study. Turk J Urol. 2022;48(3):201–208. 10.5152/tud.2022.21326 35634938 PMC9730261

[54] Oh SJ . Current surgical techniques of enucleation in holmium laser enucleation of the prostate. Invest Clin Urol. 2019;60(5):333–342. 10.4111/icu.2019.60.5.333 PMC6722407 31501795

[55] Martos M , Katz JE , Parmar M , Jain A , Soodana‐Prakash N , Punnen S , et al. Impact of perioperative factors on nadir serum prostate‐specific antigen levels after holmium laser enucleation of prostate. BJUI Compass. 2021;2(3):202–210. 10.1002/bco2.68 35475131 PMC8988639

[56] Bhojani N , Bidair M , Kramolowsky E , Desai M , Doumanian L , Zorn KC , et al. Aquablation therapy in large prostates (80‐150 mL) for lower urinary tract symptoms due to benign prostatic hyperplasia: final WATER II 5‐year clinical trial results. J Urol. 2023;210(1):143–153. 10.1097/JU.0000000000003483 37115632 PMC12721682

[57] McVary KT . Erectile dysfunction and lower urinary tract symptoms secondary to BPH. Eur Urol. 2005;47(6):838–845. 10.1016/j.eururo.2005.02.001 15925081

[58] Sato R , Sano A , Watanabe K , Matsushita Y , Watanabe H , Tamura K , et al. Effects of changes in erectile function after holmium laser enucleation of the prostate on postoperative outcomes in patients with benign prostatic hyperplasia. In Vivo. 2022;36(6):2960–2964. 10.21873/invivo.13039 36309353 PMC9677774

[59] Jaidane M , Arfa NB , Hmida W , Hidoussi A , Slama A , Sorba NB , et al. Effect of transurethral resection of the prostate on erectile function: a prospective comparative study. Int J Impot Res. 2010;22(2):146–151. 10.1038/ijir.2009.56 19940854

[60] Donovan JL , Peters TJ , Neal DE , Brookes ST , Gujral S , Chacko KN , et al. A randomized trial comparing transurethral resection of the prostate, laser therapy and conservative treatment of men with symptoms associated with benign prostatic enlargement: the clasp study. J Urol. 2000;164(1):65–70. 10.1016/S0022-5347(05)67450-2 10840426

[61] Cheng BKC , Li TCF , Yu CHT . Sexual outcomes of endoscopic enucleation of prostate. Andrologia. 2020;52(8):e13724. 10.1111/and.13724 32557813

[62] Which anatomic structures should be preserved during aquablation contour planning to optimize ejaculatory function? A case‐control study using ultrasound video recordings to identify surgical predictors of postoperative anejaculation ‐ ScienceDirect [Internet]. [cited 2024 Jan 22]. Available from: https://www.sciencedirect.com/science/article/pii/S0090429521000868 10.1016/j.urology.2021.01.023 33482130

[63] Herrmann TRW , Gravas S , de la Rosette JJ , Wolters M , Anastasiadis AG , Giannakis I . Lasers in transurethral enucleation of the prostate—do we really need them. J Clin Med. 2020;9(5):1412. 10.3390/jcm9051412 32397634 PMC7290840

[64] Press B , Gardezi M , Kim DD , Lokeshwar S , Rahman S , Siev M , et al. Ejaculatory preserving holmium laser enucleation of the median lobe: preserving sexual function while improving urinary outcomes. Urology. 2023;173:175–179. 10.1016/j.urology.2022.12.035 36646177

[65] Manasa T , Reddy N , Puvvada S , Mylarappa P . Evaluating outcomes of combined bladder neck and supramontanal sparing ejaculatory preserving transurethral resection of the prostate: results from a prospective, randomised study. Cent Eur J Urol. 2022;75(3):292–298. 10.5173/ceju.2022.0004 PMC9628722 36381163

[66] Nguyen DD , Misraï V , Bach T , Bhojani N , Lingeman JE , Elterman DS , et al. Operative time comparison of aquablation, greenlight PVP, ThuLEP, GreenLEP, and HoLEP. World J Urol. 2020;38(12):3227–3233. 10.1007/s00345-020-03137-8 32124018

[67] Heidenberg DJ , Nethery E , Wymer KM , Judge N , Cheney SM , Stern KL , et al. Are adverse events during surgery for benign prostatic hyperplasia device related? A review of the MAUDE database. Urologia. 2024;91(2):249–255. 10.1177/03915603241240646 38520298

[68] Kaplan‐Marans E , Martinez M , Wood A , Cochran J , Dubowitch E , Schulman A . Aquablation, prostatic urethral lift, and transurethral water vapor therapy: a comparison of device‐related adverse events in a national registry. J Endourol. 2022;36(2):231–235. 10.1089/end.2021.0455 34314240

[69] Hwang EC , Jung JH , Borofsky M , Kim MH , Dahm P . Aquablation of the prostate for the treatment of lower urinary tract symptoms in men with benign prostatic hyperplasia. Cochrane Database Syst Rev. 2019;(2):CD013143, Available from:. 10.1002/14651858.CD013143.pub2/full 30759311 PMC6373984

[70] Müllhaupt G , Enzler‐Tschudy A , Horg K , Bubendorf L , Pratsinis M , Schmid HP , et al. Informative value of histological assessment of tissue acquired during aquablation of the prostate. World J Urol. 2021;39(6):2043–2047. 10.1007/s00345-020-03426-2 32902728

[71] Müllhaupt G , Güsewell S , Schmid HP , Zumstein V , Betschart P , Engeler DS , et al. Aquablation versus holmium laser enucleation of the prostate in the treatment of benign prostatic hyperplasia in medium‐to‐large‐sized prostates (ATHLETE): protocol of a prospective randomised trial. BMJ Open. 2021;11(5):e046973. 10.1136/bmjopen-2020-046973 PMC8098986 33941632

