# Supplementary material for: Comparing outcomes of Aquablation versus holmium laser enucleation of prostate in the treatment of benign prostatic hyperplasia: A network meta‐analysis
Source: BJUI Compass. 2024 Oct 30;5(12):1231–45. doi: 10.1002/bco2.454 (PMC11685169; doi:10.1002/bco2.454)
Supplement: Supplementary file 1 — Table S1. Outcomes of sensitivity analysis. Table S2. Inclusion and Exclusion criteria of studies. Table S3. GRADE ratings. Figure S1. Supplementary: IIEF‐5 outcomes comparing EF change at 3 and 12 months. [file BCO2-5-1231-s001.docx]

Supplementary information for: Comparing Outcomes of Aquablation® versus Holmium Laser Enucleation of Prostate in the Treatment of Benign Prostatic Hyperplasia: A Network Meta-Analysis

Table S1. Outcomes of sensitivity analysis

| Outcome | Time point | Mean difference (95% CI) HoLEP v. WJAT |
| --- | --- | --- |
| Qmax | 3 months | 6.22 (-0.53 to 15.70) |
|  | 12 months | 4.94 (-2.65 to 16.80) |
|  | 24 months | 2.39 (-14.50 to 24.00) |
| IPSS | 3 months | 0.41 (-13.10 to 15.60) |
|  | 12 months | 4.74 (-1.64 to 11.10) |
|  | 24 months | 4.06 (-11.40 to 19.60) |
| PVR | 3 months | 30.20 (10.30 to 52.80) |
|  | 12 months | 54.80 (30.90 to 80.40) |
|  | 24 months | 68.80 (39.60 to 112.00) |
| IPSS-QoL | 3 months | -0.99 (-6.40 to 2.48) |
|  | 12 months | -0.02 (-8.40 to 4.61) |
|  | 24 months | 0.29 (-6.51 to 11.56) |
| Resection time | NA | 40.53 (19.65 to 62.06) |
| Hb loss | NA | -1.03 (-3.37 to 1.82) |
| Hospitalization time | NA | 5.13 (-18.21 to 37.42) |
| Blood transfusion rate | NA | 0.23 (0.01 to 7.88) |
| Acute urinary retention rate | NA | 0.53 (0.08 to 5.68) |
| Serious adverse events rate | NA | 1.16 (0.22 to 11.20) |

Supplementary Table 1: Analysis results after excluding studies at high Risk of Bias as determined using the Cochrane RoB-2 tool. Comparisons that did not include studies with a high Risk of bias were not represented here.

Table S2. Inclusion and Exclusion criteria of studies

| **Authors (year)** | **N** | **Inclusion Criteria** | **Exclusion Criteria** | **Device settings** |
| --- | --- | --- | --- | --- |
| Gilling et al (2017) | 181 | Age 45-80 yo  PV 30-80 g  IPSS ≥ 12  Qmax < 15 mL/s | H/o prostate or bladder cancer, neurogenic bladder, bladder stone or clinically significant bladder diverticulum, active UTI, chronic prostatitis, urethral stricture, meatal stenosis or BNC, damaged external urinary sphincter, stress urinary incontinence  PVR >300 mL or urinary retention  Use of self-catheterization,  Prior prostate surgery  Anticoagulants, bladder anticholinergics or with severe cardiovascular disease | AQUABEAM System (PROCEPT BioRobotics, USA) |
| Wilson et al (2006) | 61 | PV 40–200 g  IPSS ≥ 8  Qmax ≤ 15 ml/s  PVR < 400 ml  Schaffer grade ≥ 2 | Prostate Cancer  Catheterised patients  H/o previous urethral or prostatic surgery | 60-100W Ho laser (Lumenis, Israel) with a maximum average power of 100 W (2J at 50Hz). Wavelength: 2,140 nm with maximum power was set at 100 W for each case |
| Rigatti et al (2005) | 100 | Age ≤ 75 yo  PV 30-100 g  Qmax ≤ 15 ml/s  PVR ≤ 100  Medical therapy failure  Schäfer grade ≥ 2  Obstruction on ICS nomogram or Abram-Griffiths number ≥ 40 | Neurogenic bladder  Diagnosis of prostate cancer  Any previous prostate, bladder neck or urethral surgery  Presence of an indwelling catheter | Holmium laser energy was delivered by a 360-um fiber placed in a 24F resectoscope. Enucleation was performed at 2J and 35 Hz |
| Fayad et al (2011) | 60 | All patients who met the inclusion criterion of presenting with LUTS due to BPH and in whom surgery was indicated were included in the study | IPSS score < 8  Qmax > 15ml/sec   Minimal PVR.  PV < 20 g  Patients with urethral stricture, neurogenic bladder, vesicoureteral reflux, huge retentive bladder diverticulum in whom open surgery to remove the diverticulum was preferred, history of urethral or prostatic surgeries, prostate cancer.  Anticoagulant therapy | 100W holmium yttrium aluminum garnet laser (Device Lumenis, Medical Systems, USA). |
| Kuntz et al (2004) | 200 | PV < 100 g  IPSS > 12  Qmax < 12 ml/ (voided volume > 150 ml)  PVR > 50 ml  Schafer grade > 2 | Prostate Cancer  Undergone previous urethral or prostatic surgery | Maximum average power of 80W (2J at 40 Hz) or 100W (2J at 50 Hz). |
| Sun et al (2013) | 164 | Age < 90 yo without contraindication to surgery  PV ≤ 100 g  IPSS ≥ 8  Qmax ≤ 10 ml/s  PVR ≤ 50 ml | Treatment with transurethral prostate surgery previously  Neurogenic bladder  Suspected prostate cancer | F26 OLYMPUS holmium laser prostate rectoscope with 550-lm holmium laser fiber was connected, and the output power was 98 W |
| Gupta et al (2005) | 150 | PV > 40 g | H/o of prostatic and urethral surgery, neurovesical dysfunction, or prostate carcinoma | 550-µm end- firing laser fiber and 100 W holmium-YAG laser source (Coherent Inc., USA). Settings: 80–100 W at 2–1.5 J/ s and 50–40 Hz. |
| Jhanwar et al (2017) | 164 | Age < 75 yo  PV > 60 g  Qmax <15 ml/s  PVR >150 ml  Gross hematuria due to BPH  Recurrent UTI or AUR  Schafer Grade > II | Neurogenic bladder  Urethral Stricture   Prostate Cancer or previous history of intervention | End firing holmium laser fiber (550 µm, versa power suit) with power setting (2 J at 40–50 Hz, 80–100 W). |
| Bašić et al (2013) | 40 | PV up to 50cc  IPSS > 19  PVR = 50ml,  Recurrent AUR or indwelling urinary catheter  Recurrent UTI or hematuria due to BPH | Voiding disorders out of BPH origin  Previous urethral, bladder neck or prostatic surgery, and  H/o prostate cancer | 2J/50Hz, 100-watt holmium laser device (VersaPulse® PowerSuite, Lumenis, Israel), using a 550-m end Firing fiber (SlimLineTM 550, Lumenis Inc.) |
| Gilling et al (2017) | 21 | PV 25-80 mL  IPSS >12   Qmax <12 ml/s,  Schafer scale ≥ 216 | Active UTI, Abnormal renal function, or elevated PSA,   Urinary retention or PVR ≥400 ml,  H/o lower urinary tract surgery or suspected prostate/bladder cancer,  Neurogenic bladder and/or external urinary sphincter abnormalities,  Previous prostate surgery, current therapy affecting prostate physiology  Medical condition that would pose an unacceptable patient risk. | AquaBeam™ (PROCEPT BioRobotics, USA) |
| Bach et al (2018) | 118 | NA | Anticoagulation therapy other than Aspirin 100 mg | AquaBeam™ (PROCEPT BioRobotics, USA) |
| Desai et al (2018) | 47 | Age 50-80 yo  PV 20-120 g  IPSS >12  Qmax ≤ 15 ml/s  H/o inadequate  response, contraindication to or refusal of medical therapy. | H/o prostate/bladder cancer, prostate surgery, or Elevated PSA  Neurogenic bladder,  Prostatitis within the last year, active infection,  Urethral stricture, meatal stenosis or BNC,  Use of anticoagulants, or gross hematuria,  Allergy to device materials,  Use of immune suppressants or corticosteroids, and serious medical or mental illness | Second generation AQUA BEAM system |
| Yee et al (2022) | 20 | Age 50-70 yo  Refractory to medical treatment with catheterization | Active UTI or on anticoagulation;  Bladder pathology including bladder stone and bladder cancer;  Confirmed neurological pathology that would alter their detrusor or sphincter function  Prior surgical intervention to the prostate or prostate cancer. | Aquabeam system (Procept BioRobotics, USA) |
| Labban et al (2021) | 59 | Refractory to medical treatment | Denied to patients on anticoagulation (except patients on Aspirin 100 mg which was stopped five days prior to the procedure) | Aquabeam system (Procept BioRobotics, USA) |
| Helfand et al (2021) | 34 | PV > 150 mL | NA | AquaBeam System (Procept BioRobotics, USA) |
| Misrai et al (2019) | 30 | Age 45–80 yo  PV 30–80 g  IPSS ≥ 12  Refractory to medical treatment | H/o prostate or bladder cancer; Neurogenic bladder, bladder stones  Urethral stricture, meatal stenosis, BNC or external urinary sphincter injury,  Active infection or prostatitis in the last year,  Qmax > 15 ml/s and/or PVR >300 ml,  H/o catheterisation in the last 14 days or use of intermittent catheterisation  Daily use of anticoagulants or aspirin that could not be stopped,  Use of bladder-acting medications, illicit substance use, or other medical or psychiatric conditions that could prevent study follow-up or potentially confound the results | Aquabeam system (Procept BioRobotics, USA) |
| Whiting et al (2020) | 55 | PV 20-150 g | H/o gross haematuria, known coagulopathy or platelet disorders  Use of systemic immunosuppressants, or any severe illness that would prevent complete study participation or confound results  Contraindication to both general and spinal anaesthesia  Patients unable to stop anticoagulants, antiplatelets, NSAIDs. Participants unwilling to accept a blood transfusion | Aquabeam system (Procept BioRobotics, USA) |
| De Cillis et al (2022) | 60 | PV < 80 ml  IPSS ≥ 10  Qmax ≤12 ml/s  BPH-related LUTS | Prostate cancer diagnosis, previous prostate surgery, or Prostatic calcifications  Indwelling catheter or self-catheterization  Urethral stenosis, bladder stones, clinically significant bladder diverticulum,   Anticoagulants or on antiplatelet therapy that could not be discontinued | Aquabeam system (Procept BioRobotics, USA) |
| Desai et al, India (2017) | 101 | - 45 to 80 years of age - prostate volume 80 -150 g - IPSS ≥ 12 - Qmax < 15 mL/s, - a serum creatinine <2 mg/dL, - a history of inadequate or failed response to medical   therapy  mental capability and willingness to participate in the study | - body mass index ≥ 42, - a history of prostate or bladder cancer, - clinically significant bladder   calculus or bladder diverticulum,   - active infection, - previous urinary tract surgery, - urinary catheter use - daily for 90 or more days consecutively, - chronic pelvic pain, - diagnosis of urethral stricture, meatal   stenosis or bladder neck contracture,   - use of anticholinergic agents specifically for bladder problems,   other general conditions that could prevent adequate study follow-up | the AQUABEAM System (PROCEPT BioRobotics, Redwood  Shores, California, USA) |
| Becker et al, UK (2017) | 54 | IPSS ≥ 12  Qmax ≤ 15 ml/s  Refractory to medical therapy | Previous urethral/prostatic surgery  Prostate cancer  Urethral strictures and neurogenic bladder | HoLEP was performed using a 2080-nm pulsed Ho:YAG laser (Auriga ® XL, Boston Scientific, Germany) at 39.6-W (2.2 Joule, 18 Hz). |
| Gilling et al, New Zealand (2007) | 71 | IPSS ≥ 8  Qmax < 15 ml/s  PVR < 400 ml  Schaffer grade ≥ 2 | Catheterized patients  Previous prostatic or urethral surgery | 100-W holmium laser (VersaPulse, Lumenis, Israel). |
| Vavassori et al., Italy (2007) | 330 | NA | NA | Pulsed, high-powered, 60–80W holmium neodymium: yttrium-aluminum-garnet laser was preferred until 2002, when it was upgraded to  100W |
| Abdel Hakim et al., Egypt (2009) | 230 | NA | NA | High power pulsed 100 W VersaPulse holmium laser unit (Lumenis, USA) |

Acute Urinary Retention; BNC: Bladder Neck Contracture; BPH: Benign Prostatic Hyperplasia; BPO: Benign Prostatic Obstruction; HoLEP: Holmium Laser Enucleation of the Prostate; H/o: History of; ICS: International Continence Society; IPSS: International Prostate Symptom Score; LUTS: Lower Urinary Tract Symptoms ; NSAIDs: Nonsteroidal Anti-Inflammatory Drugs; PVR: Post-Void Residual; PV: Prostate Volume; Qmax: Maximum Flow Rate; UTI: Urinary Tract Infection

Table S3: GRADE ratings

| *Outcomes* | Timeframe | RCT only | Prospective single arm studies only | |
| --- | --- | --- | --- | --- |
|  |  | Overall rating | Overall rating |  |
| IPSS | 3mo | Moderate ^a^ | Low ^d^ |  |
| IPSS | 12 mo | Moderate ^a^ | Low ^d^ |  |
| IPSS | 24-36 mo | Moderate ^a^ | Low ^d^ |  |
| IPSS | 60 mo | Moderate ^a^ | NA |  |
|  |  |  |  |  |
| QoL | 3mo | Moderate ^a^ | Low ^d^ |  |
| QoL | 12 mo | Moderate ^a^ | NA |  |
| QoL | 24 mo | Moderate ^a^ | NA |  |
| QoL | 60 mo | Moderate ^a^ | NA |  |
|  |  |  |  |  |
| Qmax | 3mo | High * | Low ^e^ |  |
|  | 12mo | Moderate ^a^ | Low ^e^ |  |
|  | 24mo | Moderate ^a^ | Very low ^d^ |  |
|  | 60mo | Low^c^ | NA |  |
|  |  |  |  |  |
| PVR | 3mo | High * | Low ^e^ |  |
|  | 12 mo | High * | Low ^e^ |  |
|  | 24 mo | Moderate ^a^ | NA |  |
|  |  |  |  |  |
| Stricture | NA | Low ^a^ | Very low ^d^ |  |
|  |  |  |  |  |
| Incontinence | NA | Very low ^c^ | Very Low ^d^ |  |
|  |  |  |  |  |
| AUR | NA | Very low ^c^ | Very Low ^d^ |  |
|  |  |  |  |  |
| IIEF-5 | 3mo | Low ^a^ | NA |  |
|  | 12 mo | Low ^a^ | NA |  |
|  |  |  |  |  |
| Post-operative Hb Loss | - | Moderate ^b^ | Low ^e^ |  |
|  |  |  |  |  |
| Hospitalization time | - | Moderate ^b^ | Low ^e^ |  |
|  |  |  |  |  |
| Catheterization duration | - | Moderate ^b^ | Very Low ^d^ |  |
|  |  |  |  |  |
| Operative time | - | Moderate ^b^ | Low ^e^ |  |
|  |  |  |  |  |
| Blood transfusion rate | - | Low ^c^ | Low ^e^ |  |
|  |  |  |  |  |
| SAE | - | Low ^b^ | NA |  |
|  |  |  |  |  |
| RTR | - | Low ^b^ | NA |  |

Legend: Explanation for GRADE ratings:

^*^: Downgraded for High risk of bias and upgraded for upgraded for absolute effect size.

^a^ Downgraded one level each for High risk of bias and Imprecision, upgraded for absolute effect size.

^b^ Downgraded one level for High risk of bias.

^c^ Downgraded one level for High risk of bias and two levels for imprecision.

^d^ Downgraded one level (starting from low quality of evidence due to single arm trials) due to imprecision.

^e^ Low quality of evidence due to single arm trials. No downgrading of evidence from this level.

Figure 1 Supplemetary: IIEF-5 outcomes comparing EF change at 3 and 12 months


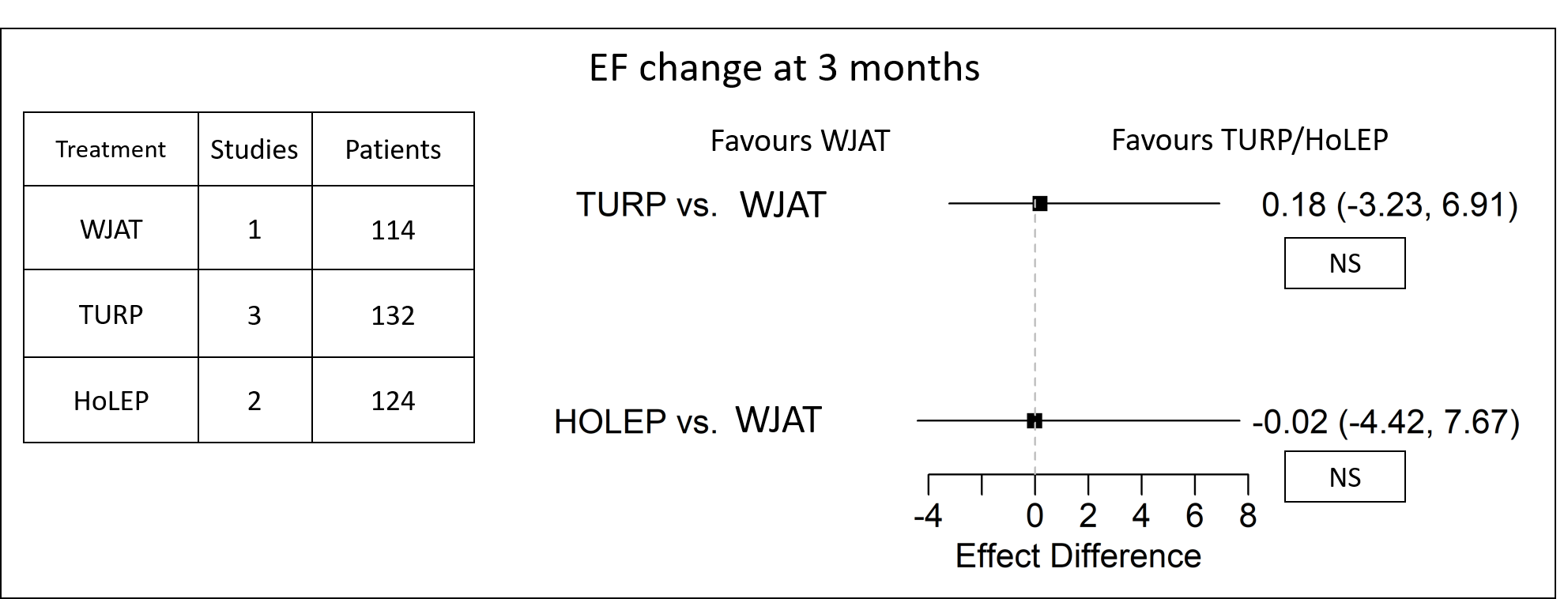

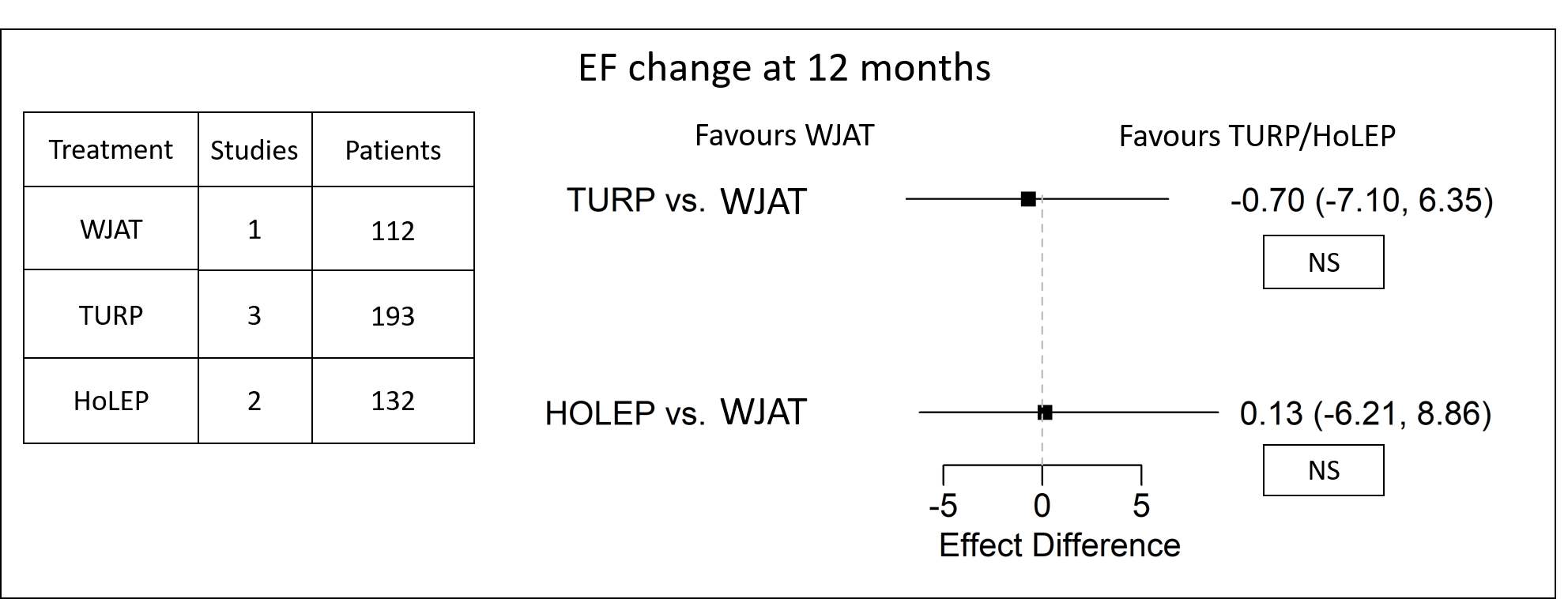


**RCT only comparison – Erectile Function (IIEF-5)**

Search strategy: Cochrane library

Holep: ("prostatic hyperplasia" OR benign prostatic hyperplasia OR "LUTS" OR "Lower urinary tract symptoms" OR "Urinary tract symptoms") AND ("Laser enucleation" OR "HoLEP" OR "enucleation of prostate" OR "Prostate enucleation" OR "Holium Laser Enucleation" OR "Prostate laser enucleation" OR "Holium Laser Enucleation of prostate") in all text

Aquablation- (aquablat* OR "robotic waterjet ablat*" OR "waterjet ablation therapy" OR WJAT OR "robotic waterjet ablation therapy") AND ("prostatic hyperplasia" OR benign prostatic hyperplasia OR "LUTS" OR "Lower urinary tract symptoms" OR "Urinary tract symptoms")

Pubmed: HoLEP search

- (("Laser enucleation"[All fields] OR "HoLEP"[All fields] OR "enucleation of prostate"[All fields] OR "Prostate enucleation"[All fields] OR "Holmium Laser Enucleation"[All fields] OR "Prostate laser enucleation"[All fields] OR "Holmium Laser Enucleation of prostate")) AND ((("prostatic hyperplasia"[MeSH Terms] OR benign prostatic hyperplasia[Text Word] OR "LUTS" OR "Lower urinary tract symptoms" OR "Urinary tract symptoms")))

Aquablation - (aquablat* OR "robotic waterjet ablat*" OR "waterjet ablation therapy" OR WJAT OR "robotic waterjet ablation therapy") AND ("prostatic hyperplasia" OR benign prostatic hyperplasia OR "LUTS" OR "Lower urinary tract symptoms" OR "Urinary tract symptoms")
